# Loss of Bacterial Cell Pole Stabilization in Caulobacter crescentus Sensitizes to Outer Membrane Stress and Peptidoglycan-Directed Antibiotics

**DOI:** 10.1128/mBio.00538-20

**Published:** 2020-05-05

**Authors:** Simon-Ulysse Vallet, Lykke Haastrup Hansen, Freja Cecillie Bistrup, Signe Aagaard Laursen, Julien Bortoli Chapalay, Marc Chambon, Gerardo Turcatti, Patrick H. Viollier, Clare L. Kirkpatrick

**Affiliations:** a Department of Microbiology and Molecular Medicine, Institute of Genetics and Genomics in Geneva (iG3), Faculty of Medicine/CMU, University of Geneva, Geneva, Switzerland; b Department of Biochemistry and Molecular Biology, University of Southern Denmark, Odense, Denmark; c Biomolecular Screening Facility, School of Life Sciences, Ecole Polytechnique Fédérale de Lausanne (EPFL), Lausanne, Switzerland; Emory University School of Medicine

**Keywords:** antibiotic resistance, *Caulobacter crescentus*, TonB-dependent receptor, cell envelope, cell polarity, vancomycin

## Abstract

Rod-shaped bacteria frequently localize proteins to one or both cell poles in order to regulate processes such as chromosome replication or polar organelle development. However, the roles of polar factors in responses to extracellular stimuli have been generally unexplored. We employed chemical-genetic screening to probe the interaction between one such factor from Caulobacter crescentus, TipN, and extracellular stress and found that TipN is required for normal resistance of cell envelope-directed antibiotics, including vancomycin which does not normally inhibit growth of Gram-negative bacteria. Forward genetic screening for suppressors of vancomycin sensitivity in the absence of TipN revealed the TonB-dependent receptor ChvT as the mediator of vancomycin sensitivity. Loss of ChvT improved resistance to vancomycin and cefixime in the otherwise sensitive Δ*tipN* strain. The activity of the two-component system regulating ChvT (ChvIG) was increased in Δ*tipN* cells relative to the wild type under some, but not all, cell wall stress conditions that this strain was sensitized to, in particular cefixime and detergent exposure. Together, these results indicate that TipN contributes to cell envelope stress resistance in addition to its roles in intracellular development, and its loss influences signaling through the ChvIG two-component system which has been co-opted as a sensor of cell wall stress in *Caulobacter*.

## Abstract (precis)

Maintenance of an intact cell envelope is essential for free-living bacteria to protect themselves against their environment. In the case of rod-shaped bacteria, the poles of the cell are potential weak points in the cell envelope due to the high curvature of the layers and the need to break and reform the cell envelope at the division plane as the cells divide. We have found that TipN, a factor required for correct division and cell pole development in Caulobacter crescentus, is also needed for maintaining normal levels of resistance to cell wall-targeting antibiotics such as vancomycin and cefixime, which interfere with peptidoglycan synthesis. Since TipN is normally located at the poles of the cell and at the division plane just before cells complete division, our results suggest that it is involved in stabilization of these weak points of the cell envelope as well as its other roles inside the cell.

## INTRODUCTION

The asymmetrically dividing alphaproteobacterium Caulobacter crescentus (hereafter *Caulobacter*) temporally and spatially regulates its cell cycle in order to propagate the correct positioning of the polar stalk and flagellum from one generation of cells to the next generation (1–4). Among the regulatory factors that transmit positional information differentiating the cell poles from the rest of the cell body is the TipN polarity factor that localizes to the division plane and remains at the new pole once division is completed (5, 6). This provides a molecular marker that defines the newest cell pole (i.e., the one arising from the most recent cell division) and ensures that the flagellum assembly factors are subsequently recruited there. Cells lacking TipN have a flagellum placement defect, but they also have other deficiencies. For example, cells lacking TipN are defective in chromosome partitioning, likely due to loss of an interaction between TipN and ParA which is required for prompt and unidirectional segregation of the *parS*-ParB complex from one cell pole to the other after initiation of chromosome replication (7, 8), a phenotype unrelated to flagellum positioning. Therefore, TipN acts as a new-pole-specific multifunctional regulator that spatially integrates at least two vital cellular processes (cellular differentiation and chromosome partitioning).

If the correct function of TipN depends on its relocalization from flagellar pole to division plane at cytokinesis, how is this achieved? It is currently unknown what signal triggers delocalization of TipN away from the pole and its reassembly at the division septum, but it has been shown that TipN can interact with the septum-located Tol-Pal complex (9). This complex regulates later stages of cell division and is required for connecting the inner membrane, peptidoglycan, and outer membrane during the peptidoglycan remodeling that is necessary for septum formation between the two daughter cells. It is conceivable, although unproven, that the arrival of Tol-Pal at the division plane is the recruitment signal for TipN. Tol-Pal is required for outer membrane fission at cell division and remains at the newly divided cell pole (in complex with TipN) after separation of the daughter cells is complete. Unlike the situation in Escherichia coli, the *Caulobacter* Tol-Pal complex is essential (10), and it has been proposed that it is required for general cell envelope integrity in addition to carrying out the last stage of cell division (9). Although TipN has not been shown to participate directly in maintenance of cell wall integrity, a genetic link between TipN and other cell envelope-located protein complexes (in addition to Tol-Pal) has been identified. We previously found that loss of function of TipN was synthetically lethal with the quinolone antibiotic nalidixic acid (Nal) and discovered that this was because Nal induces strong expression of an efflux pump of the RND (resistance-nodulation-cell division) family, named AcrAB2-NodT, while the normal target of Nal (DNA gyrase) was apparently unaffected and that this overexpression was specifically intolerable to Δ*tipN* cells (11). Deletion of the efflux pump increased, rather than decreased, Nal resistance, and seemed to be related to pump structure rather than function because a chemical inhibitor of efflux (phenylalanine arginine β-naphthylamide [PAβN]) did not increase Nal resistance. However, the molecular mechanism by which TipN protects against the Nal-induced overexpression of this efflux pump is still not known.

In Gram-negative bacteria that live in oligotrophic environments (such as *Caulobacter*), the outer membrane is equipped with systems to (i) detect and respond negatively to potential toxins or (ii) detect and respond positively to nutrients. The *Caulobacter* genome encodes 105 putative signal transduction proteins and 65 TonB-dependent receptors (12), which are potentially important for environmental sensing and nutrient uptake, respectively. The TonB-dependent receptors have not been systematically characterized, but individual members of this family have been found to be specific receptors for vitamins or metal ions (13–16) or to be transcriptionally upregulated under conditions of carbon or nitrogen starvation (17). Regulation of TonB-dependent receptors, as for outer membrane proteins in general, is often posttranscriptionally controlled by small noncoding RNAs (sRNAs) (18). The *Caulobacter* genome encodes 133 sRNAs (19), of which only a few have been characterized, but in these cases, they appear to be components of stress-response signaling pathways (20, 21). The sRNA ChvR was recently shown to downregulate a single TonB-dependent receptor of unknown function, ChvT, in response to acid stress, starvation, or DNA damage (22). ChvR transcription under these conditions was induced by the two-component system ChvIG, which is highly conserved among the alphaproteobacteria and particularly important for the association of pathogenic (23, 24) or symbiotic (25) alphaproteobacterial species with their hosts. However, in *Caulobacter*, it seems to have been repurposed to sense other types of environmental stress, since this species does not associate with a host cell.

In this study, we sought to clarify why high expression of an efflux pump should be deleterious to Δ*tipN* cells and whether it was possible to identify other environmental conditions which were similarly toxic to this strain to pinpoint the molecular mechanism of TipN’s role in protection against AcrAB2-NodT overexpression. We show that the toxicity of the efflux pump overexpression arises from general intolerance of the Δ*tipN* strain to overexpression of this class of cell envelope-spanning systems and can also be elicited by heterologous overexpression of other efflux pump components. Moreover, by high-throughput chemical-genetic screening, we found that this strain is also sensitized to cell wall-targeting antibiotics, particularly vancomycin, and that the vancomycin sensitivity can be suppressed by loss of the TonB-dependent receptor ChvT. We observed activation of the ChvIG-induced promoter of ChvT’s negative regulator *chvR* during cell envelope stress conditions, including efflux pump overexpression, presence of cell wall-targeted antibiotics, and the detergent sodium deoxycholate. The Δ*tipN* strain was sensitized to detergent stress relative to the wild-type strain, and the detergent-induced activity of *chvR* promoter activity in Δ*tipN* cells was correspondingly higher. Overall, these findings indicate a previously unsuspected role for TipN in stabilization of the cell envelope of *Caulobacter* and that the absence of TipN can influence cell wall stress signaling mediated by the ChvIG-ChvR-ChvT pathway.

## RESULTS

### TipN is required for maintenance of normal growth, cell morphology, and division under AcrAB2-NodT-mediated cell envelope stress.

**FIG 1:**
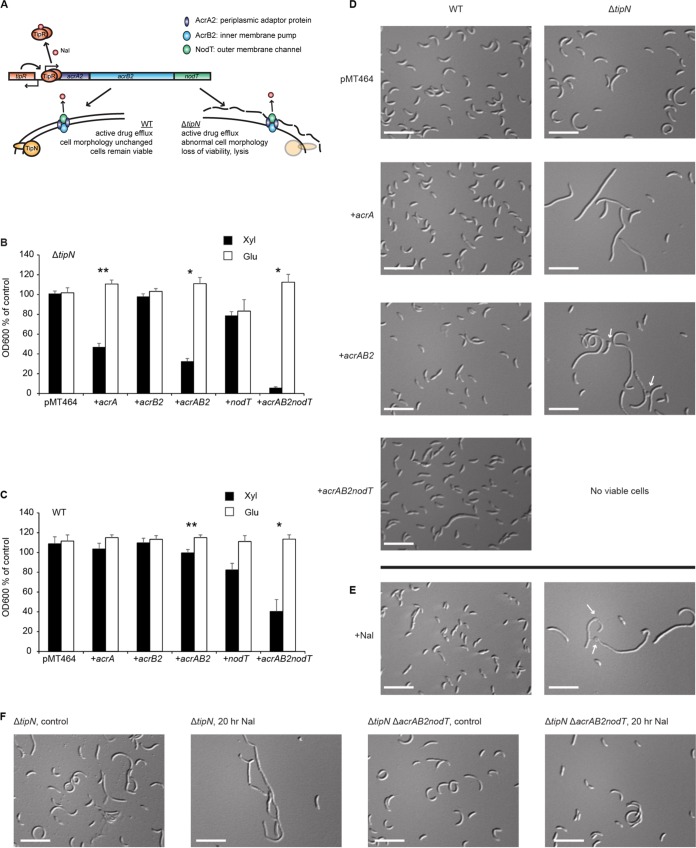
Nalidixic acid exposure and *acrAB2-nodT* overexpression cause identical cell morphology defects in Δ*tipN* cells. (A) Graphical model of the regulatory effect of nalidixic acid (Nal) on the *acrAB2-nodT* operon and its consequences in WT and Δ*tipN* strains. Nalidixic acid causes the repressor TipR to leave the promoter, resulting in high expression of the efflux pump genes and their incorporation into the cell envelope. In cells lacking TipN, this results in growth inhibition and abnormal cell morphology. (B) Growth assay of Δ*tipN* cells with empty vector pMT464 and a series of pMT464 overexpression plasmids containing *acrAB2-nodT* component genes separately or together, treated with 0.3% xylose or 0.2% glucose for 20 h before culture density measurement. Data are expressed as a percentage of the OD_600_ of control cultures of each strain in untreated PYE medium. All graphs show means ± standard errors of the means (SEM) from three independent experiments unless otherwise stated in the figure legends. (C) Growth assay of WT cells with empty vector pMT464 and a series of pMT464 overexpression plasmids containing *acrAB2-nodT* component genes separately or together, treated with 0.3% xylose or 0.2% glucose for 20 h before culture density measurement. Data are expressed as a percentage of the OD_600_ of control cultures of each strain in untreated PYE. Statistical significance is denoted by asterisks (*, *P* < 0.05; **, *P* < 0.01) in pairwise (Xyl versus Glu) comparisons. (D) Differential interference contrast (DIC) images of WT and Δ*tipN* cells containing pMT464, pMT464-*acrA*, pMT464-*acrAB2*, or pMT464-*acrAB2-nodT* and treated with 0.3% xylose for 20 h before imaging. Control cultures of both strains treated with 0.2% glucose were prepared and imaged in every biological replicate and had identical cell morphology to the empty vector control cultures. (E) DIC images of WT and Δ*tipN* cells treated with 20 μg/ml Nal for 20 h before imaging. (F) DIC images of Δ*tipN* and Δ*tipN* Δ*acrAB2-nodT* cells treated with 20 μg/ml Nal or not treated with Nal for 20 h before imaging. White arrows indicate sites of cell bulging and bleb formation. Bars, 5 μm.

We previously identified an AcrAB family multidrug efflux pump module, *acrAB2-nodT*, that was strongly induced in response to Nal and whose induction was responsible for the Δ*tipN* mutant’s Nal sensitivity (11) (Fig. 1A). However, at this stage, we wished to confirm that AcrAB2-NodT overexpression was solely sufficient for the effect. We therefore made expression constructs using a vector carrying the xylose-inducible promoter upstream of the individual *acrA*, *acrB2*, or *nodT* genes, *acrAB2*, or the whole operon. With this construct, expression levels in the absence of xylose (inducer) or presence of glucose (repressor) were similar to basal expression from the native promoter, while xylose-induced expression from this construct was comparable to Nal-induced expression from the native promoter (see Fig. S1A in the supplemental material). In the Δ*tipN* mutant, xylose-induced overexpression of *acrA*, *acrAB2*, or *acrAB2-nodT* inhibited growth of the cells compared to the glucose-treated control (Fig. 1B), which was not observed in the wild type (WT) (Fig. 1C). Consistent with a nonspecific effect, we observed that the Δ*tipN* mutant was sensitized to heterologous overexpression of the *acrA3* (*Caulobacter*) and *mexA* (Pseudomonas aeruginosa) genes, encoding the periplasmic adaptor proteins which connect the inner membrane efflux pump to the outer membrane channel, relative to the WT (Fig. S1B). Both WT and Δ*tipN* strains were sensitized to overexpression of the *acrAB3*, E. coli *acrAB*, and *Pseudomonas mexAB* gene pairs (Fig. S1B and C), suggesting that heterologous overexpression of foreign (or endogenous but not usually strongly expressed) complexes of periplasmic adaptor together with inner membrane pump components is not well tolerated in *Caulobacter*. The decreased growth seen in the endpoint measurements corresponded to the cultures entering stationary phase at a lower optical density at 600 nm (OD_600_) while the growth rate in exponential phase was not affected (Fig. S2). However, the *acrAB2-nodT* operon was not intrinsically toxic when overexpressed to comparable levels (Fig. S1D) in a heterologous (E. coli) host, even in a strain with compromised outer membrane stability (*lptD* allele formerly known as *imp4213* [26]) (Fig. S1E). These results suggest that the sensitivity of the Δ*tipN* mutant to Nal is independent of nonspecific Nal-mediated toxicity and show that the Δ*tipN* mutant is more sensitive than the WT to overproduction of individual efflux pump components in general, suggesting that it is susceptible to nonspecific overload of the cell envelope unrelated to drug efflux activity.

10.1128/mBio.00538-20.1 FIG S1 Xylose-induced overexpression of the *acrAB2-nodT* genes in *trans* is comparable to expression from the endogenous promoter and is toxic to Caulobacter crescentus but not E. coli. (A) Immunoblotting with specific antibody against AcrA of cell extracts of NA1000 (WT) or Δ*acrAB2-nodT* containing the empty vector pMT464 or the pMT464-*acrAB2nodT* overexpression vector in which expression of the cloned genes is induced with 0.3% xylose (or repressed by 0.2% glucose) for 3 h before cell harvesting. The same strains were also treated with 20 μg/ml nalidixic acid for 3 h before harvesting in order to directly compare nalidixic acid induction from the native promoter to xylose-induced overexpression from the pMT464 promoter in the same gel. Cell cultures were normalized by OD_600_ before harvesting to ensure equivalent loading in all lanes of the gel. Details of AcrA protein purification, polyclonal antibody generation, and Western blotting are given in Text S1 in the supplemental material. (B) Growth assay of Δ*tipN* cells with empty vector pMT464 and the heterologous overexpression constructs for induced overexpression of *Caulobacter acrA*(*B*)*3*, E. coli *acrA*(*B*), and P. aeruginosa *mexA*(*B*), treated with 0.3% xylose or 0.2% glucose for 20 h before culture density measurement. Data are expressed as a percentage of the OD_600_ of control cultures of each strain in untreated PYE. (C) Growth assay of WT cells with empty vector pMT464 and the heterologous overexpression constructs for induced overexpression of *Caulobacter acrA*(*B*)*3*, E. coli *acrA*(*B*), and P. aeruginosa *mexA*(*B*), treated with 0.3% xylose or 0.2% glucose for 20 h before culture density measurement. Data are expressed as a percentage of the OD_600_ of control cultures of each strain in untreated PYE. Statistical significance is denoted by * for *P* < 0.05 and ** for *P* < 0.01 in pairwise (Xyl versus Glu) comparisons. (D) Immunoblotting with specific antibody against AcrA in cell extracts prepared from WT *Caulobacter* treated with or without 20 μg/ml nalidixic acid for 3 h before harvesting, alongside E. coli cell extracts containing empty vector pSRK-Km or the overexpression construct pSRK-*acrAB2nodT*, with or without 1 mM IPTG for 3 h before harvesting. Cell cultures were normalized by OD_600_ before harvesting to ensure equivalent loading in all lanes of the gel. (E) Viability assay of WT E. coli (TB28) and *lptD* mutant with empty vector pSRK-Km or the overexpression construct pSRK-*acrAB2nodT* with or without 1 mM IPTG in the preculture on LB agar with 1 mM IPTG. Starter cultures were diluted into LB with or without 1 mM IPTG and grown to exponential phase before harvesting, normalizing by OD_600_, diluting (serial 10-fold dilutions), and plating 5-μl spots of the diluted cells. Representative images of two biological replicates are shown. Download FIG S1, TIF file, 1.9 MB. Copyright © 2020 Vallet et al. 2020 Vallet et al. This content is distributed under the terms of the Creative Commons Attribution 4.0 International license.

10.1128/mBio.00538-20.2 FIG S2 AcrAB2-NodT overexpression causes Δ*tipN* cultures to prematurely enter stationary phase. Growth curves of WT and Δ*tipN* strains with empty vector control (pMT464) or AcrAB2-NodT overexpression plasmid (+ABN) grown either in nonsupplemented PYE or in PYE with 0.3% xylose to induce overexpression in 96-well plate format at 30°C for 48 h. Control cultures containing 0.2% glucose (represses the xylose-inducible promoter) were also performed in all replicates of the experiment and always grew better (to a higher stationary-phase OD_600_) than cultures in PYE alone. Download FIG S2, TIF file, 1.1 MB. Copyright © 2020 Vallet et al. 2020 Vallet et al. This content is distributed under the terms of the Creative Commons Attribution 4.0 International license.

To investigate how efflux pump overexpression could be exerting its toxic effect, we studied cell morphology of WT and Δ*tipN* strains overexpressing the *acrAB2-nodT* efflux pump (whole operon or individual components) from the xylose-inducible promoter. Compared to the empty vector control, Δ*tipN* cells overexpressing the *acrA* gene became filamentous, while overexpressing the *acrAB2* genes in Δ*tipN* caused bulging, branching, and frequent lysis in addition to filamentation (Fig. 1D), similar to Δ*tipN* cells exposed to Nal (Fig. 1E). WT cells had mild bulging and filamentation defects with Nal and with *acrAB2-nodT* operon overexpression, while overexpression of the *acrA* or *acrAB2* constructs alone had no effect. To confirm that the Nal-induced cell morphology defect was due to efflux pump overexpression from its native promoter, we imaged identically treated Δ*tipN* and Δ*tipN* Δ*acrAB2-nodT* cells, and as expected, the deletion of the *acrAB2-nodT* pump genes protected Δ*tipN* cells from the Nal-induced morphology defect (Fig. 1F). Together, these experiments suggest that TipN is required for maintenance of correct cell division during cell envelope overload with periplasmic proteins or complexes.

### Chemical genetic screening reveals that Δ*tipN* cells are also sensitized to cell wall-targeting antibiotics independent of AcrAB2-NodT.

**FIG 2:**
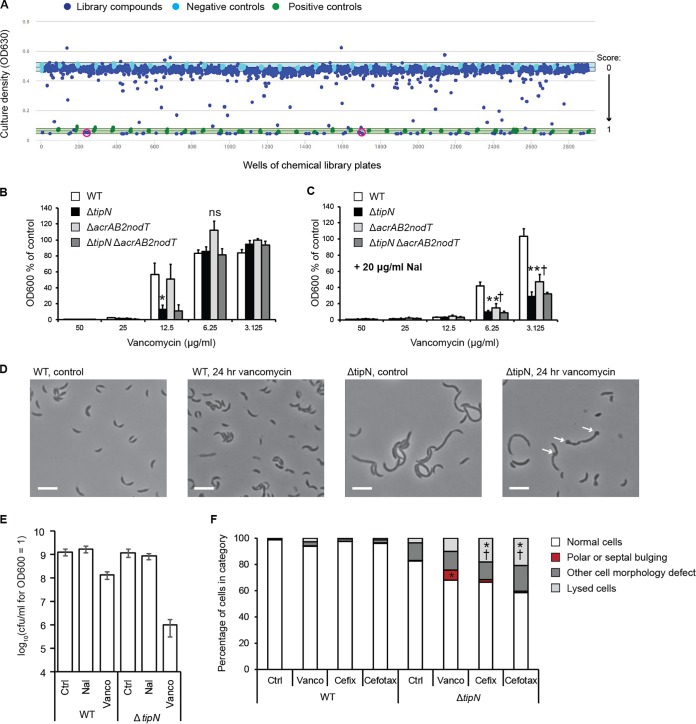
Nal and Vanco are synergistically lethal. (A) Scatterplot of the results of screening WT Caulobacter crescentus against the Prestwick Chemical Library for Nal-dependent growth inhibitors (all library compounds at 10 μM and Nal at 20 μg/ml under all conditions, including the controls). The vertical axis indicates culture growth (measured by OD_630_). and the horizontal axis denotes the number of wells. Blue points correspond to wells containing library compounds, green points are the positive-control wells (2 μg/ml = 6.25 μM chloramphenicol), and light blue points are negative controls containing DMSO at 0.1% (final concentration of the diluent of the library compounds; does not inhibit growth). The light blue band behind the negative-control points corresponds to mean ± 3 standard deviations around the mean of the negative controls. The *Z*’ score for this screen was 0.894, and the *Z*’ score for the corresponding control screen, of WT cells without Nal, was 0.820. Pink circles denote the wells containing vancomycin (Vanco). Vanco had a similarly close score to those of the positive controls in the Δ*tipN* screen (0.88) as it did in the Nal dependence screen shown here (1.00). (B) Growth assay of WT, Δ*tipN*, Δ*acrAB2-nodT*, and Δ*tipN* Δ*acrAB2-nodT* cultures treated with Vanco at the indicated concentrations for 20 h before culture density measurement. Data are expressed as a percentage of the OD_600_ of control cultures of each strain in untreated PYE medium. (C) Growth assay of WT, Δ*tipN*, Δ*acrAB2-nodT*, and Δ*tipN* Δ*acrAB2-nodT* cultures treated with Vanco at the indicated concentrations and 20 μg/ml Nal for 20 h before culture density measurement. Data are expressed as a percentage of the OD_600_ of control cultures of each strain in untreated PYE. Statistical significance is denoted by the following symbols: * for *P* < 0.05 and ** for *P* < 0.01 in the comparison of the WT strain to the Δ*tipN* strain; † for *P* < 0.05 in the comparison of the WT strain to the Δ*acrAB2-nodT* mutant (“ns” signifies “not statistically significant”). (D) Phase-contrast images of WT and Δ*tipN* cells treated with 15 μg/ml Vanco or not treated with Vanco for 24 h before imaging. White arrows indicate bleb formation sites at the poles and division plane of cells. Bars, 5 μm. (E) Calibration of CFU/ml of WT and Δ*tipN* cultures grown in untreated medium or in medium with 20 μg/ml Nal or 15 μg/ml Vanco for 24 h and normalized by OD_600_. (F) Quantitative microscopy of WT and Δ*tipN* cells grown in untreated medium or medium with 15 μg/ml Vanco, 5 μg/ml cefixime, or 5 μg/ml cefotaxime for 24 h before imaging and classified by cell morphology. Data were gathered from three independent biological replicates and are expressed as the average percentage of the total number of cells counted per replicate. The total numbers of cells counted per condition (combined from all three replicates) were as follows: WT control, 848; WT Vanco, 902; WT cefixime, 931; WT cefotaxime, 900; Δ*tipN* control, 765; Δ*tipN* Vanco, 1,126; Δ*tipN* cefixime, 679; Δ*tipN* cefotaxime, 807. Statistical significance is denoted by the following symbols: * for *P* < 0.05 in comparing drug-treated culture to control Δ*tipN* culture; † for *P* < 0.05 in comparing treatment with Vanco and treatment with the other two drugs.

Having established that the Δ*tipN* mutant is susceptible to cell wall stress caused by overload of proteins located in the cell envelope, specifically efflux pump components, we then investigated whether we could identify any sensitivity to small-molecule stressors that mimicked this effect by chemical genetic screening. We screened WT *Caulobacter* against the Prestwick Chemical Library in the presence and absence of Nal, reasoning that any drugs that could inhibit growth in the presence of Nal but not in its absence would be synthetically toxic with Nal-induced AcrAB2-NodT overexpression. They would therefore act as a chemical phenocopy of the Δ*tipN* mutant phenotype, in which case their mechanism(s) of action could indicate why TipN is protective against cell wall stress as described above. After identifying the compounds that fitted this criterion, we compared the list of hits from this screen against those that had been identified as Δ*tipN*-specific growth inhibitors in a previous study (27). This was done in order to differentiate hits that were synergistic with Nal but unrelated to its activity as a transcriptional inducer of AcrAB2-NodT expression from hits that were likely to be synergistic with Nal-induced AcrAB2-NodT expression, in order to minimize false-positive results. Strikingly, the compound that showed the strongest Δ*tipN*-dependent and Nal-dependent growth inhibition was vancomycin (Vanco) (Fig. 2A). We also identified the third-generation cephalosporin cefixime as a hit (albeit a low-scoring hit) which fitted these criteria and noted that a structurally similar drug, cefotaxime, had been a low-scoring hit in the Δ*tipN-*specific screen although it did not show Nal-dependent growth inhibition in WT (Fig. S3). Together, these results suggested that the Δ*tipN* mutation could cause cell envelope instability that allows access of cell wall-targeting drugs to the periplasm and sites of peptidoglycan synthesis. To validate the screening data, we performed growth assays to test the dose dependence of Vanco sensitivity in WT and Δ*tipN* cells and whether the Δ*acrAB2-nodT* deletion could protect Δ*tipN* cells from Vanco as it did from Nal. Consistent with the screening experiment, the Δ*tipN* mutant was sensitized to Vanco relative to the WT at 12.5 μg/ml (in the high-throughput screen, Vanco was present at 10 μM = 15 μg/ml) (Fig. 2B), and the addition of Nal sensitized all strains still further (Fig. 2C). Decreased endpoint growth measurements corresponded to entry into stationary phase at a lower OD_600_ (Fig. S4A). However, the Δ*acrAB2-nodT* deletion did not protect the WT or Δ*tipN* strain against the synergistic effect of Nal plus Vanco, which would have been expected if Vanco had acted as a true phenocopy of the Δ*tipN* mutant phenotype at the genetic as well as phenotypic level. Therefore, although the presence of Nal, and presumably the subsequent *acrAB2-nodT* overexpression, had been sufficient to cause increased Vanco sensitivity in WT cells, the Vanco sensitivity was not fully dependent on *acrAB2-nodT* as it had been for the Δ*tipN* mutant’s Nal sensitivity. Imaging of WT and Δ*tipN* cells after 24-h Vanco treatment showed that WT cells were not affected at the level of cell morphology, while the Δ*tipN* strain showed frequent signs of lysis, especially blebbing at the cell poles and division plane (where TipN is normally located in WT cells) which we had only infrequently observed in Nal-treated Δ*tipN* cells (compare Fig. 2D to Fig. 1E and F). Calculation of surviving CFU/milliliter after Nal and Vanco treatment and calibration to OD_600_ values showed that the Nal-induced abnormal morphology did not influence the OD_600_ measurement or the viability of the cells, while Vanco treatment reduced viability by 1 log unit in WT cultures but 3 log units in Δ*tipN* (Fig. 2E). Finally, we quantified the proportion of cells with abnormal morphology after treatment with Vanco, cefixime, or cefotaxime and found that Δ*tipN* cultures treated with Vanco had a significantly higher proportion, 7.6%, of cells showing bulging or blebs at the poles or division plane compared to Δ*tipN* control or WT cultures under any condition (<1%) or Δ*tipN* cultures treated with the other two drugs (<2%) (Fig. 2F). Although we did not observe more frequent polar or septal bulging with cefixime or cefotaxime, the Δ*tipN* cultures exposed to these drugs had significantly more lysed cells than control or Vanco-treated Δ*tipN* cells. Hence, although the chemical-genetic screening results had initially indicated that the Nal and Vanco toxicity to the Δ*tipN* strain were linked, our follow-up validation experiments have shown that Nal and Vanco exert distinct cell envelope-directed toxic effects in the absence of TipN, presumably through independent pathways, and that TipN appears to stabilize the cell poles against Vanco.

10.1128/mBio.00538-20.3 FIG S3 High-throughput chemical-genetic screening suggests that Δ*tipN* cells are also sensitive to the cephalosporin antibiotics cefixime and cefotaxime. (A) Scatterplot of the same screening data shown in Fig. 4A of WT Caulobacter crescentus (NA1000) grown in the presence of 20 μg/ml Nal in addition to the Prestwick Chemical Library compounds, with the points corresponding to cefixime wells (purple) and cefotaxime wells (turquoise) indicated. (B) Scatterplot of Δ*tipN* cells screened against the Prestwick Chemical Library with the points corresponding to cefixime wells (purple) and cefotaxime wells (turquoise) indicated. This screening experiment was previously published (27); its *Z*’ score was 0.894. Download FIG S3, TIF file, 1.6 MB. Copyright © 2020 Vallet et al. 2020 Vallet et al. This content is distributed under the terms of the Creative Commons Attribution 4.0 International license.

10.1128/mBio.00538-20.4 FIG S4 *chvT* deletion does not improve the growth of Δ*tipN* in Nal or during overexpression of the AcrAB2-NodT efflux pump. (A) Growth curves of WT, Δ*tipN*, Δ*acrAB2-nodT*, Δ*tipN* Δ*acrAB2-nodT*, Δ*chvT*, and Δ*tipN* Δ*chvT* strains in PYE with and without 15 μg/ml Vanco in 96-well plate format at 30°C for 48 h. (B) Growth assay of Δ*tipN*, Δ*tipN* Δ*chvT*, Δ*tipN chvT*::*2v15*, and Δ*tipN chvT*::*3l15* strains treated with 20 μg/ml Nal, 15 μg/ml Vanco, or both antibiotics together for 20 h before culture density measurement. Data are expressed as a percentage of the OD_600_ of control cultures of each strain in untreated PYE. (C) Growth assay of Δ*tipN* Δ*chvT* cells with empty vector pMT464 and a series of pMT464 overexpression plasmids containing *acrAB2-nodT* component genes separately or together, treated with 0.3% xylose or 0.2% glucose for 20 h before culture density measurement. Data are expressed as a percentage of the OD_600_ of control cultures of each strain in untreated PYE. Download FIG S4, TIF file, 1.1 MB. Copyright © 2020 Vallet et al. 2020 Vallet et al. This content is distributed under the terms of the Creative Commons Attribution 4.0 International license.

### Vancomycin sensitivity of the Δ*tipN* strain is suppressed by loss of the TonB-dependent outer membrane receptor ChvT.

**FIG 3:**
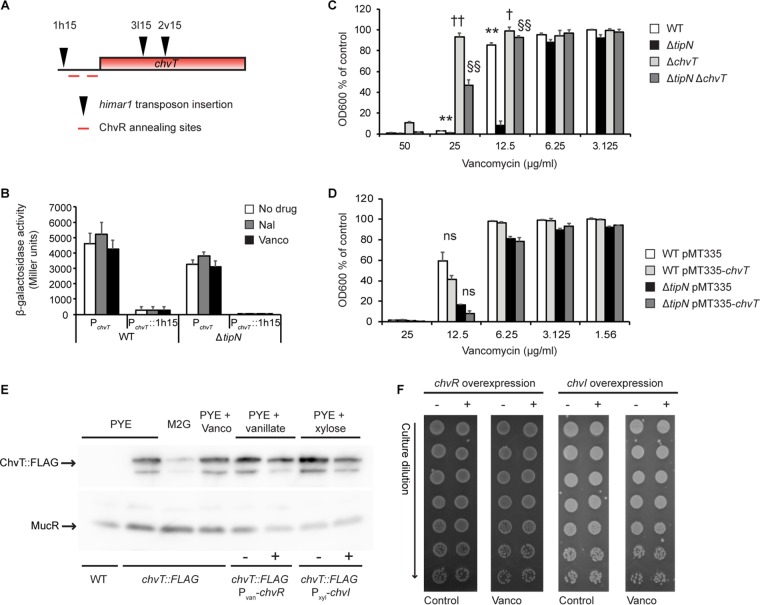
Loss of ChvT confers Vanco resistance. (A) Positions (approximately to scale) of the 1h15, 2v15, and 3l15 *himar1* transposon insertions in the *chvT* coding region or promoter that conferred Vanco resistance to the Δ*tipN* mutant. (B) Promoter activity of P*_chvT_* (WT promoter or mutant promoter containing the 1h15 *himar1* transposon insertion) in WT and Δ*tipN* strains in untreated PYE medium or medium with 20 μg/ml Nal or 15 μg/ml Vanco for 3 h. (C) Growth assay of WT, Δ*tipN*, Δ*chvT*, and Δ*tipN* Δ*chvT* strains treated with Vanco at the indicated concentrations for 20 h before culture density measurement. Data are expressed as a percentage of the OD_600_ of control cultures of each strain in untreated PYE medium. Statistical significance is indicated as follows: ** for *P* < 0.01 in comparing the WT to Δ*tipN* strains, † for *P* < 0.05 and †† for *P* < 0.01 in comparing the WT to Δ*chvT* strains, and §§ for *P* < 0.01 in comparing the Δ*tipN* to Δ*tipN* Δ*chvT* strains. (D) Growth assay of WT and Δ*tipN* containing the overexpression plasmid pMT335-*chvT* or the pMT335 empty vector, treated with 50 μM vanillate and Vanco at the indicated concentrations for 20 h before culture density measurement. Data are expressed as a percentage of the OD_600_ of control cultures of each strain in PYE containing 50 μM vanillate only (“ns” signifies “not statistically significant”). (E) Western blot of ChvT::3xFLAG and MucR (constitutively expressed loading control) in cells grown under the indicated conditions for 24 h and normalized by OD_600_ before harvesting. Vanco was used at 15 μg/ml, vanillate at 50 μM, and xylose at 0.3%. For the *chvT*::*3xFLAG* P_van_-*chvR* strains, − indicates the empty vector pBVMCS-6, and + indicates the ChvR overexpression plasmid pKF382. For the *chvT*::*3xFLAG* P_xyl_-*chvI* strains, − indicates the empty vector pMT464, and + indicates the ChvI overexpression plasmid pMT464-*chvI*. Images are representative of three independent biological replicates. (F) Efficiency of plating assay of WT *Caulobacter* containing the ChvR or ChvI overexpression plasmid or corresponding empty vector controls with or without Vanco (15 μg/ml). The – and + symbols signify empty vector or overexpression plasmid, respectively, as in the legend to panel E. For the ChvR overexpression strains, the plates also contained chloramphenicol and 50 μM vanillate. For the ChvI overexpression strains, the plates also contained kanamycin and 0.3% xylose. Images are representative of three independent experiments.

To search for genetic suppressors of the Δ*tipN* Vanco sensitivity phenotype, we performed forward genetic screening by constructing a pooled transposon mutant library in the Δ*tipN* strain and enriching it in Vanco-containing medium for individual clones that were capable of forming single colonies on media with 15 μg/ml Vanco. From this screen, we mapped three independent transposon insertions in or near the *CCNA_03108* gene (Fig. 3A), annotated as a TonB-dependent outer membrane receptor of unknown substrate specificity named ChvT (22). Two transposon insertions (2v15 and 3l15) mapped to the coding sequence, while one (1h15) was located in the promoter region. Cloning of the *chvT* promoter region from the WT strain and the 1h15 transposon mutant into a β-galactosidase transcriptional reporter showed that (i) the 1h15 insertion in P*_chvT_* abolished transcriptional activity, (ii) the promoter was active in both Δ*tipN* and WT strains, and (iii) it did not respond to the presence of either Nal or Vanco (Fig. 3B). Therefore, the promoter insertion is likely a loss-of-function allele.

In support of our hypotheses that (i) loss of function of *chvT* protects against Vanco and (ii) the Vanco and Nal sensitivity phenotypes are unlinked, the Δ*tipN* Δ*chvT* double deletion mutant grew better in Vanco similarly to the 3l15 transposon insertion strain, but neither of these was protected against Nal, and the combination of Nal and Vanco was still highly toxic to all strains, suggesting that the two antibiotics can exert a synergistic effect that the Δ*chvT* mutant cannot defend against (Fig. S4B). The Δ*chvT* deletion also protected WT *Caulobacter* against high concentrations of Vanco, showing that it is not specifically associated with the Δ*tipN* genotype (Fig. 3C). However, overexpression of *chvT* did not cause any significant sensitivity to Vanco in either the WT or Δ*tipN* strain (Fig. 3D), indicating that it is the presence or absence of ChvT that dictates the phenotype and that upregulating ChvT does not influence it. We performed the same experiments with cefixime and cefotaxime and confirmed that the Δ*tipN* mutant is sensitized to both of these antibiotics but found that the Δ*chvT* deletion was less effective in protection against cefixime and cefotaxime than it had been for Vanco, and *chvT* overexpression had no effect on growth in the presence of these antibiotics (Fig. S5). We also tested whether Δ*chvT* could protect the Δ*tipN* mutant against overexpression of *acrAB2-nodT* from the xylose-inducible promoter, and consistent with its lack of effect against Nal, we did not observe any change relative to the Δ*tipN* single mutant (compare Fig. S4C to Fig. 1B). Therefore, loss of ChvT protects the Δ*tipN* mutant against cell wall stress when induced by Vanco, and to a lesser extent against cefixime and cefotaxime, but not against cell wall stress caused by other factors.

10.1128/mBio.00538-20.5 FIG S5 *chvT* deletion protects against cefixime and cefotaxime in addition to vancomycin, but *chvT* overexpression does not increase sensitivity to these antibiotics. (A) Growth assay of WT, Δ*tipN*, Δ*chvT*, and Δ*tipN* Δ*chvT* strains treated with cefixime at the indicated concentrations for 20 h before culture density measurement. Data are expressed as a percentage of the OD_600_ of control cultures of each strain in untreated PYE. (B) Growth assay of WT and Δ*tipN* strains containing the overexpression plasmid pMT335-*chvT* or the pMT335 empty vector and treated with 50 μM vanillate and cefixime at the indicated concentrations for 20 h before culture density measurement. Data are expressed as a percentage of the OD_600_ of control cultures of each strain in PYE containing 50 μM vanillate only. (C) Growth assay of WT, Δ*tipN*, Δ*chvT*, and Δ*tipN* Δ*chvT* strains treated with cefotaxime at the indicated concentrations for 20 h before culture density measurement. Data are expressed as a percentage of the OD_600_ of control cultures of each strain in untreated PYE. (D) Growth assay of WT and Δ*tipN* strains containing the overexpression plasmid pMT335-*chvT* or the pMT335 empty vector and treated with 50 μM vanillate and cefotaxime at the indicated concentrations for 20 h before culture density measurement. Data are expressed as a percentage of the OD_600_ of control cultures of each strain in PYE containing 50 μM vanillate only. Statistical significance in panels A and C is denoted as follows: * for *P* < 0.05 and ** for *P* < 0.01 in comparing the WT to Δ*tipN* strain, † for *P* < 0.05 in comparing the WT to the Δ*chvT* strain, and § for *P* < 0.05 in comparing the Δ*tipN* strain to the Δ*tipN* Δ*chvT* strain. Download FIG S5, TIF file, 0.2 MB. Copyright © 2020 Vallet et al. 2020 Vallet et al. This content is distributed under the terms of the Creative Commons Attribution 4.0 International license.

It was recently shown that ChvT is the sole target of the sRNA ChvR, which binds to the 5′ untranslated region (UTR) of the *chvT* mRNA and downregulates ChvT production (22). To assess whether steady-state levels of ChvT were altered during vancomycin treatment, we treated cells carrying a *chvT*::*3xFLAG* allele with Vanco and compared ChvT::3xFLAG steady-state levels with cells grown in minimal (M2G) medium or carrying plasmids to overexpress either ChvR or the response regulator ChvI. Growth in M2G medium reduced the ChvT::3xFLAG levels in the cells, consistent with the overexpression of ChvR sRNA under this condition (22), but Vanco treatment had no effect (Fig. 3E). Overexpression of ChvR or ChvI partially reduced ChvT::3xFLAG levels (Fig. 3E), but this reduction had no effect on Vanco resistance (Fig. 3F). Taken together, these data indicate that ChvT production does not respond to the presence of Vanco and that any amount of ChvT in the cells confers Vanco sensitivity; only the *chvT* deletion is sufficient to confer resistance.

### The Δ*tipN* strain displays elevated ChvIG-ChvR-ChvT signaling activity during detergent or cephalosporin antibiotic stress conditions.

**FIG 4:**
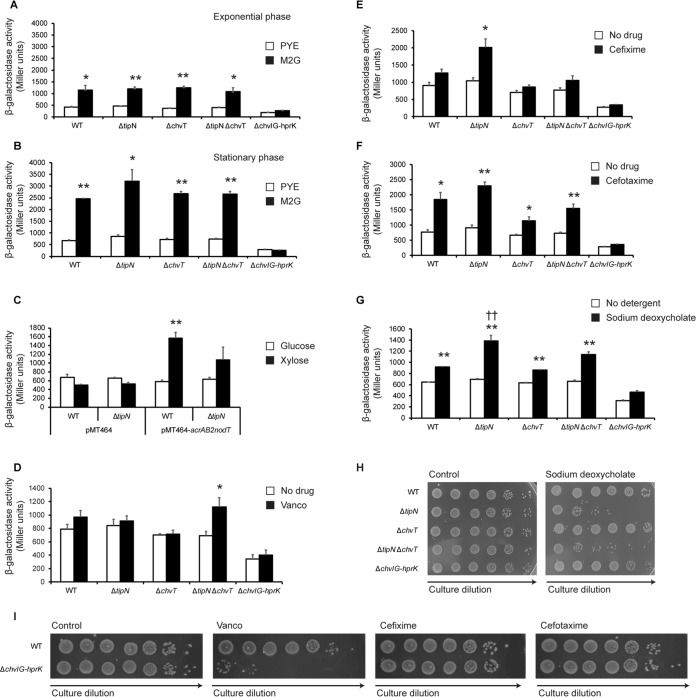
The ChvIG-ChvR-ChvT signaling pathway responds to cell wall stress in *Caulobacter*. (A) Promoter activity of P*_chvR_* in WT, Δ*tipN*, Δ*chvT*, Δ*tipN* Δ*chvT*, and Δ*chvIG-hprK* strains in exponential phase (3-h growth after inoculation) in PYE or M2G medium. (B) Promoter activity of P*_chvR_* in WT, Δ*tipN*, Δ*chvT*, Δ*tipN* Δ*chvT*, and Δ*chvIG-hprK* strains in stationary phase (24-h growth after inoculation) in PYE or M2G medium. These experiments were performed independently of those measured in panel A and are not derived from repeat measurements of the same cultures. (C) Promoter activity of P*_chvR_* in WT and Δ*tipN* strains containing overexpression plasmid pMT464-*acrAB2nodT* or empty vector pMT464 and treated with 0.3% xylose or 0.2% glucose for 24 h. (D) Promoter activity of P*_chvR_* in WT, Δ*tipN*, Δ*chvT*, Δ*tipN* Δ*chvT*, and Δ*chvIG-hprK* strains with or without 15 μg/ml Vanco for 24 h. (E) Promoter activity of P*_chvR_* in WT, Δ*tipN*, Δ*chvT*, Δ*tipN* Δ*chvT* , and Δ*chvIG-hprK* strains with or without 5 μg/ml cefixime (= 10 μM) for 24 h. (F) Promoter activity of P*_chvR_* in WT, Δ*tipN*, Δ*chvT*, Δ*tipN* Δ*chvT*, and Δ*chvIG-hprK* strains with or without 5 μg/ml cefotaxime (= 10 μM) for 24 h. (G) Promoter activity of P*_chvR_* in WT, Δ*tipN*, Δ*chvT*, Δ*tipN* Δ*chvT*, and Δ*chvIG-hprK* strains with or without 0.6 mg/ml sodium deoxycholate for 24 h. (H) Viability assay of WT, Δ*tipN*, Δ*chvT*, Δ*tipN* Δ*chvT*, and Δ*chvIG-hprK* strains with or without 0.6 mg/ml sodium deoxycholate for 24 h under the same conditions as in panel G. (I) Efficiency of plating assay of WT and Δ*chvIG-hprK* strains on untreated PYE medium or medium with 15 μg/ml Vanco, 5 μg/ml cefixime, or 5 μg/ml cefotaxime. Images are representative of three independent experiments. Statistical significance is indicated as follows: * for *P* < 0.05 and ** for *P* < 0.01 in within-strain comparisons (all panels, control versus test condition) and †† for *P* < 0.01 for between-strain comparison under the same experimental condition (WT versus Δ*tipN* strains both with sodium deoxycholate [panel G only]).

Since loss of ChvT restored Vanco resistance to the Δ*tipN* strain, we hypothesized that the signaling pathway leading to transcription of ChvR, which is mediated by the two-component system ChvIG, could be altered in Δ*tipN* cells. Since it is known to respond to external factors such as starvation and acid stress, both in *Caulobacter* (22) and in other alphaproteobacteria in which ChvIG is conserved (28), we investigated whether it also responds to the cell wall-targeting drugs identified in our chemical screen or to other sources of cell wall stress. We constructed a transcriptional P*_chvR_*-*lacZ* reporter and tested its activity in WT, Δ*tipN*, Δ*chvT*, Δ*tipN* Δ*chvT*, and Δ*chvIG-hprK* strains (this acts as a negative control for P*_chvR_* activity, as the two-component system ChvIG is the only known regulator of *chvR* transcription and therefore should display no or minimal P*_chvR_* activity). We measured the activity of this promoter in exponential and stationary phase in rich (PYE) and minimal (M2G) media to assess whether the promoter activity of this construct increased under conditions that upregulated ChvR RNA levels. Consistent with existing data (22) and with our ChvT::3xFLAG Western blotting (Fig. 3E), there was very low promoter activity in exponential phase in PYE medium, while it became active in M2G medium (Fig. 4A). However, we also observed increased promoter activity in stationary phase in PYE as well as in M2G (Fig. 4B), indicating that this promoter starts to become active at later stages of growth even in rich medium. Having validated that our reporter responded as expected to the M2G control condition, we tested whether it was also affected by overexpression of *acrAB2-nodT*. Xylose-induced *acrAB2-nodT* overexpression caused increased activity of P*_chvR_* compared to the glucose or empty vector controls in both WT and Δ*tipN* strains (Fig. 4C), showing that the ChvIG-*chvR* signaling pathway can respond to loading of the cell envelope with AcrAB2-NodT efflux pump complexes. We then investigated the effects of Vanco, cefixime, and cefotaxime on the P*_chvR_* promoter to see if it also responded to exogenous, as well as endogenous, cell wall stress. Vancomycin did not induce promoter activity in any of the strains except the Δ*tipN* Δ*chvT* double mutant (Fig. 4D). However, the cephalosporin antibiotics did induce promoter activity in a strain-dependent manner. Cefixime provoked increased P*_chvR_* activity only in the Δ*tipN* strain (Fig. 4E). Cefotaxime increased its activity in all the strains, although the magnitude of the increase was lower in the Δ*chvT* and Δ*tipN* Δ*chvT* mutants than in the WT and Δ*tipN* strains (Fig. 4F). This increase was always ChvIG dependent, as it was never seen in the Δ*chvIG-hprK* strain. Finally, we investigated the effect of inducing outer membrane stress on P*_chvR_* activity with the detergent sodium deoxycholate (16). This condition increased P*_chvR_* activity in all strains, with a significantly larger increase seen in the Δ*tipN* strain than in the WT (Fig. 4G). This increased activity correlated with a loss of cell viability in the Δ*tipN* strain compared to WT cells (Fig. 4H). Since we had observed ChvIG-dependent activation of P*_chvR_* in response to cell wall stressors, we then tested whether the Δ*chvIG-hprK* mutant showed altered sensitivity to cell wall-directed antibiotics. Indeed, this strain was highly sensitized to Vanco compared to WT (Fig. 4I), although not to cefixime or cefotaxime. In sum, our data argue in favor of a novel structural role for TipN in stabilizing the cell envelope at the weak points of poles and division plane, in addition to its developmental roles in cellular differentiation and replication, and show that the ChvIG-dependent signaling pathway in *Caulobacter* has been co-opted as a cell envelope stress sensor.

## DISCUSSION

**FIG 5:**
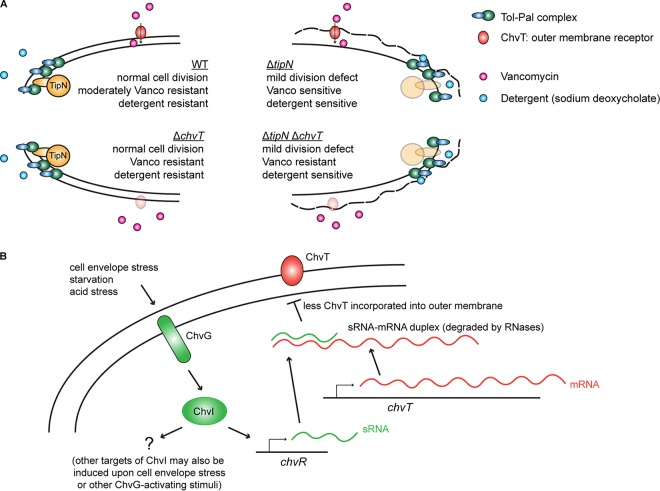
Loss of cell pole stabilization by TipN sensitizes cells to vancomycin- and detergent-induced cell wall stress. (A) Summary graphical model of the relative effects of loss of TipN and/or ChvT on sensitivity to vancomycin and sodium deoxycholate in Caulobacter crescentus. The physical interaction between TipN and the Tol-Pal complex is known from previous work (9). For the purposes of this figure, ChvT is illustrated at the lateral side of the cell, but it is likely to be distributed in multiple locations around the outer membrane (40). (B) The ChvIG-ChvR-ChvT signaling pathway of C. crescentus and its response to cell envelope stress. The biochemical details of the ChvR-*chvT* interaction and its dependence on ChvIG induced transcription of ChvR were characterized by Fröhlich et al. (22).

In the present study, we have demonstrated that the loss of TipN in *Caulobacter* is associated with sensitivity to some types of cell envelope perturbation, either by overload of cell envelope-located multidrug efflux pumps (surprisingly, since these are otherwise beneficial to the cell), destabilization of the outer membrane by detergents, or exposure to antibiotics that target the cell envelope (Fig. 5A). This work also explains our previous observation that Δ*tipN* cells treated with the efflux inhibitor PAβN grew even worse than vehicle-treated control cultures (11), as this compound is known to have nonspecific destabilizing effects on the outer membrane as well as blocking efflux (29). Since the subcellular location of TipN is restricted for the majority of the cell cycle to the new pole of the cell or to the division plane, it follows that if TipN is stabilizing the cell envelope against stress in some way, it is doing so at these places specifically. Our quantitative microscopy data support this hypothesis in the case of Vanco stress, due to the observation of increased frequency of polar or septal blebs at these locations in Vanco-treated Δ*tipN* cells compared to Vanco-treated WT cells. We did not observe the same phenotype in cefixime- or cefotaxime-treated Δ*tipN* cells, but in this case we observed significantly more lysed cells in the population. Since the polar or septal blebs we observed would have been a transient state that the cells would not remain in for long before they lysed, and the incubation period with the drugs in this experiment was 24 h, it is also possible that cefixime and cefotaxime have the same effect as Vanco but on a faster time scale. However, since the effects of the *chvT* and *chvIG* deletions on Vanco, cefixime, and cefotaxime resistance were different, our data do not exclude the possibility that these drugs use different access routes to travel to their sites of action in the peptidoglycan and/or the possibility that the presence or absence of TipN affects their access in different ways.

Our forward genetic screen for suppressors of exogenous cell wall stress (Vanco) sensitivity in the Δ*tipN* strain revealed that growth in Vanco is improved by loss-of-function mutation of the TonB-dependent receptor *chvT*. Since this was observed in both WT and Δ*tipN* backgrounds, it seems unlinked to any of the intracellular functions of TipN and is more likely to arise from the absence of ChvT making the cells’ outer membrane less permeable to the drug. However, this raises several questions about the normal function of ChvT. On the basis of homology to other TonB-dependent receptors, we predict that ChvT is a gated channel that requires energy to open and bring in the substrate, not a pore that allows free diffusion, and that it should have some degree of substrate specificity. It could be speculated on the basis of the association of *chvT* with growth in and adaptation to nutrient-rich conditions that it has specificity for peptide-based substrates and that Vanco, being a glycopeptide antibiotic, is toxic because it is similar enough to the natural substrate of ChvT that it is taken up by accident. If this were the case, it would also explain why *chvT* deletion protected the cells so much more strongly against Vanco than it did against cefixime and cefotaxime, despite these antibiotics all sharing the same target.

We have shown, using a transcriptional reporter for the promoter of the ChvR sRNA, that it can be transcriptionally activated by cell wall-targeting antibiotics (in a strain-dependent manner) and by nonspecific destabilization of the outer membrane by the detergent sodium deoxycholate, in addition to the known stimulus of M2G minimal medium (Fig. 4). Since the only known transcriptional activator of ChvR is the two-component system ChvIG, and our results show that the increases in ChvR promoter activity in response to all stimuli were dependent on ChvIG, our data support a model where the sensor kinase ChvG senses cell envelope stress and activates the response regulator ChvI to induce transcription of its target genes, including *chvR*, which would result in posttranscriptional downregulation of ChvT (Fig. 5B). However, although *chvT* is the only target of ChvR, *chvR* is highly unlikely to be the only target of ChvI. Other genes of the ChvI regulon may be more important for cell wall stress resistance, while posttranscriptional downregulation of ChvT could be a minor aspect of the response, serving to reduce the permeability of the outer membrane to exogenous sources of cell wall stress that can use ChvT as their route of entry. The ChvI regulon has been defined in Sinorhizobium meliloti and contains many genes involved in symbiosis with the host plant cells, notably including genes that regulate cell envelope integrity (30). Defining the *Caulobacter* ChvI regulon would clarify to what extent ChvI-dependent gene regulation patterns are shared or divergent between symbiotic and free-living alphaproteobacteria and whether maintenance of cell wall integrity has taken over as the major role of ChvIG signaling in *Caulobacter*. Many responses to external stress factors in *Caulobacter* are mediated by ECF (extracytoplasmic function) family σ factors (31–33), and it will be intriguing to discover how ChvIG-mediated gene expression pathways are integrated with σ factor-dependent regulation. The question how the sensor kinase ChvG of *Caulobacter* can sense and integrate such different classes of signals such as nutrient starvation, acid stress and both exogenous and endogenous sources of cell wall stress remains open. However, the orthologous sensor kinase ExoS of S. meliloti is influenced by the periplasmic regulatory factor ExoR which affects ChvI-dependent gene expression through physical interaction with ExoS (34). Similar factors may also contribute to ChvIG signaling activation in *Caulobacter*, perhaps as dedicated transducers of cell wall stress signals to ChvG.

Free-living bacteria depend on the effectiveness of their signal transduction systems to communicate the presence of external stress factors to the genetic regulatory machinery and to alter gene expression, whether at the transcriptional or posttranscriptional level, in order to adapt and overcome them. We present evidence here that the two-component system ChvIG, exclusively associated until now with host-associated (pathogenic or symbiotic) alphaproteobacteria, is also important for oligotrophic free-living bacteria as a signal transduction mechanism for alerting the bacteria to cell envelope stress. We further show that the system can modulate its activity in the event of altered intrinsic sensitivity to such stresses, as in the case of Δ*tipN* mutant cells which have higher levels of ChvIG-dependent gene activation during detergent-induced outer membrane stress, to which they are more sensitive than WT cells. TipN was already known to be a multifunctional “hub” protein required for correct asymmetric differentiation and for chromosome segregation in *Caulobacter*, and we now add another role to its repertoire, of maintaining polar cell envelope integrity. The implications of the findings presented here are twofold: first, that the two-component system ChvIG may influence gene regulation in response to cell wall-targeting antibiotics, such as might be used against pathogenic species of alphaproteobacteria, and second, that resistance to such antibiotics is dependent on factors such as TipN that maintain the integrity of the cell envelope at the otherwise vulnerable points of the cell poles and division plane. The possibility follows that polarly localized factors in other rod-shaped bacteria, not limited to the alphaproteobacteria, may also play similar cell envelope-stabilizing roles and similarly function as antibiotic resistance determinants (and hold potential as drug targets).

## MATERIALS AND METHODS

### General growth conditions.

Caulobacter crescentus and E. coli strains were routinely grown in PYE at 30°C and LB at 37°C, respectively. Antibiotics for plasmid-borne selectable markers were used at the following concentrations: tetracycline at 1 μg/ml for *Caulobacter* and 10 μg/ml for E. coli, kanamycin at 20 μg/ml (solid media) or 5 μg/ml (liquid media) for *Caulobacter* and 20 μg/ml for E. coli, chloramphenicol at 2 μg/ml for *Caulobacter* and 20 μg/ml for E. coli, and gentamicin at 1 μg/ml for *Caulobacter* and 10 μg/ml for E. coli. Preparation of M2G medium, electroporation, conjugations from E. coli to *Caulobacter* and generalized transduction with ΦCr30 bacteriophage were performed as described previously (35). Stock solutions of vancomycin, cefixime, and cefotaxime (sodium salt) were prepared at 50 mg/ml in water, 25 mg/ml in dimethyl sulfoxide (DMSO), and 50 mg/ml in water, respectively. Stock solution of sodium deoxycholate was prepared at 60 mg/ml in water.

### Strain construction.

High-copy-number plasmids (pMT335, pMT464 [36], and their derivatives) were transferred into *Caulobacter* strains by electroporation, while low-copy-number plasmids and suicide plasmids (plac290 and pNPTS138 derivatives, respectively) were transferred by conjugation from E. coli S17-1 λ*pir* (37). High- and medium-copy-number plasmids (pET28a and pSRK-Km [38] derivatives) were transformed into E. coli strains by electroporation. After integration of suicide vectors by recombination through the first homology driver, secondary recombination events were induced by sucrose counterselection, and mutants carrying resulting in-frame deletions were screened for by PCR. Primer sequences and plasmid descriptions are listed in Tables S1 and S2 in the supplemental material, respectively, and strain numbers and genotypes are listed in Table S3.

10.1128/mBio.00538-20.6 TABLE S1 Primers used in this study. Download Table S1, DOCX file, 0.02 MB. Copyright © 2020 Vallet et al. 2020 Vallet et al. This content is distributed under the terms of the Creative Commons Attribution 4.0 International license.

10.1128/mBio.00538-20.7 TABLE S2 Plasmids used in this study. Download Table S2, DOCX file, 0.02 MB. Copyright © 2020 Vallet et al. 2020 Vallet et al. This content is distributed under the terms of the Creative Commons Attribution 4.0 International license.

10.1128/mBio.00538-20.8 TABLE S3 Bacterial strains used in this study. Download Table S3, DOCX file, 0.03 MB. Copyright © 2020 Vallet et al. 2020 Vallet et al. This content is distributed under the terms of the Creative Commons Attribution 4.0 International license.

### Microscopy.

Cultures for imaging were immobilized on 1% agarose (in water) slides. Differential interference contrast (DIC) images were obtained with an Axio Imager M2 microscope equipped with a 100×/1.46 oil objective and a CoolSnap HQ^2^ camera. Phase-contrast images were obtained with an inverted Olympus IX83 microscope with a Photometrics Prime scientific complementary metal-oxide-semiconductor (sCOMS) camera. All images shown are representative of three independent experiments. Quantitative cell type classification was performed on between 150 and 550 cells per condition per replicate and taken from at least two separate fields of view for each of three independent biological replicates. Cells were classified as normal morphology (normally sized stalked, swarmer, or predivisional cells), polar or septal bulging, other cell morphology defect (including filamentation in excess of the normal length of a predivisional cell, cells with multiple constrictions, bulging at places other than the cell pole or septum, or minicells) or lysed cells (appearing light gray rather than dark by phase-contrast microscopy). For each strain and condition, the number of cells of each category was calculated and plotted as a percentage of the total number of cells.

### Chemical library screening.

The Prestwick Chemical Library was dispensed into 96-well plates so as to have two wells (in different plates) per compound. Each plate contained 8 wells of negative control (DMSO), 8 wells of positive control (chloramphenicol) and 80 wells of library compounds. Saturated overnight cultures of WT cells were diluted to an optical density at 600 nm (OD_600_) of 0.001 in two 800-ml plates containing PYE medium, and Nal was added to one plate at a final concentration of 20 μg/ml. The chemical library plates were allowed to come to room temperature and then centrifuged at 2,000 rpm for 4 min. Two hundred microliters of starter culture was seeded into each well, the lids were replaced, and the plates were incubated at 30°C for 48 h at which point cells in the negative-control wells had reached stationary phase. The optical density (630 nm) was read, and the readings were compiled into a single Excel spreadsheet per screen. The person performing this experiment was blind to the position of the library compounds in the plates. For data analysis, a score was calculated for all compounds normalized to the mean of the positive-control wells as the upper limit (value of 1) and the mean of the negative-control wells as the lower limit (value of 0). Nal-dependent hits were considered as the hits whose score fell outside the boundary of 3 standard deviations from the mean of the negative controls in the screen that was performed in the presence of Nal, but not in the control screen.

### β-Galactosidase assays.

β-Galactosidase assays on strains carrying plasmid-borne transcriptional fusions of test promoters to *lacZ* were performed by the method of Miller (39) at 30°C on cells exposed to antibiotics or other inducers (as described in the main text or figure legends) for 3 h for exponential phase or 24 h for stationary phase from three independent biological replicates. Stationary-phase cultures were diluted 1/4 to obtain an OD_660_ measurement in the range of 0.1 to 0.4 before the assay was performed.

### Sodium deoxycholate resistance assay.

Sodium deoxycholate resistance was assayed as described in reference 16. Cultures of strains to be tested were diluted from saturated overnight cultures to an OD_600_ of 0.2 in PYE medium with and without sodium deoxycholate at 0.6 mg/ml and incubated at 30°C for 24 h. Culture density was measured, normalized to the OD_600_ of the least dense culture, and serially diluted (10-fold) in PYE to 10^−6^, and 5-μl portions of the dilutions were spotted onto PYE plates. The plates were imaged after 48-h growth at 30°C. Three independent biological replicates were performed.

### Statistical analysis.

Statistical significance was analyzed by paired-sample two-tailed Student’s *t* test for within-strain (xylose to glucose) comparisons (Fig. 1; also see Fig. S1 in the supplemental material) and by nonpaired equal variance two-tailed Student’s *t* test for between-strain comparisons of drug treatment (Fig. 2), growth (Fig. 2 and 3), or β-galactosidase activity (Fig. 4).

Details of plasmid construction methods, endpoint and kinetic growth measurement, CFU to OD_600_ calibration, efficiency of plating experiments, AcrA protein purification, Western blotting, transposon mutagenesis, vancomycin resistance screening, and transposon insertion mapping are in Text S1 in the supplemental material.

10.1128/mBio.00538-20.9 TEXT S1 Supplemental Methods. Details of plasmid construction, growth and plating assays, transposon mutant library construction and screening, AcrA purification, and Western blotting. Download Text S1, DOCX file, 0.03 MB. Copyright © 2020 Vallet et al. 2020 Vallet et al. This content is distributed under the terms of the Creative Commons Attribution 4.0 International license.
