# Supplementary figures and images for: Loss of Bacterial Cell Pole Stabilization in Caulobacter crescentus Sensitizes to Outer Membrane Stress and Peptidoglycan-Directed Antibiotics
Source: mBio. 2020 May 5;11(3):e00538-20. doi: 10.1128/mBio.00538-20 (PMC7403779; doi:10.1128/mBio.00538-20)

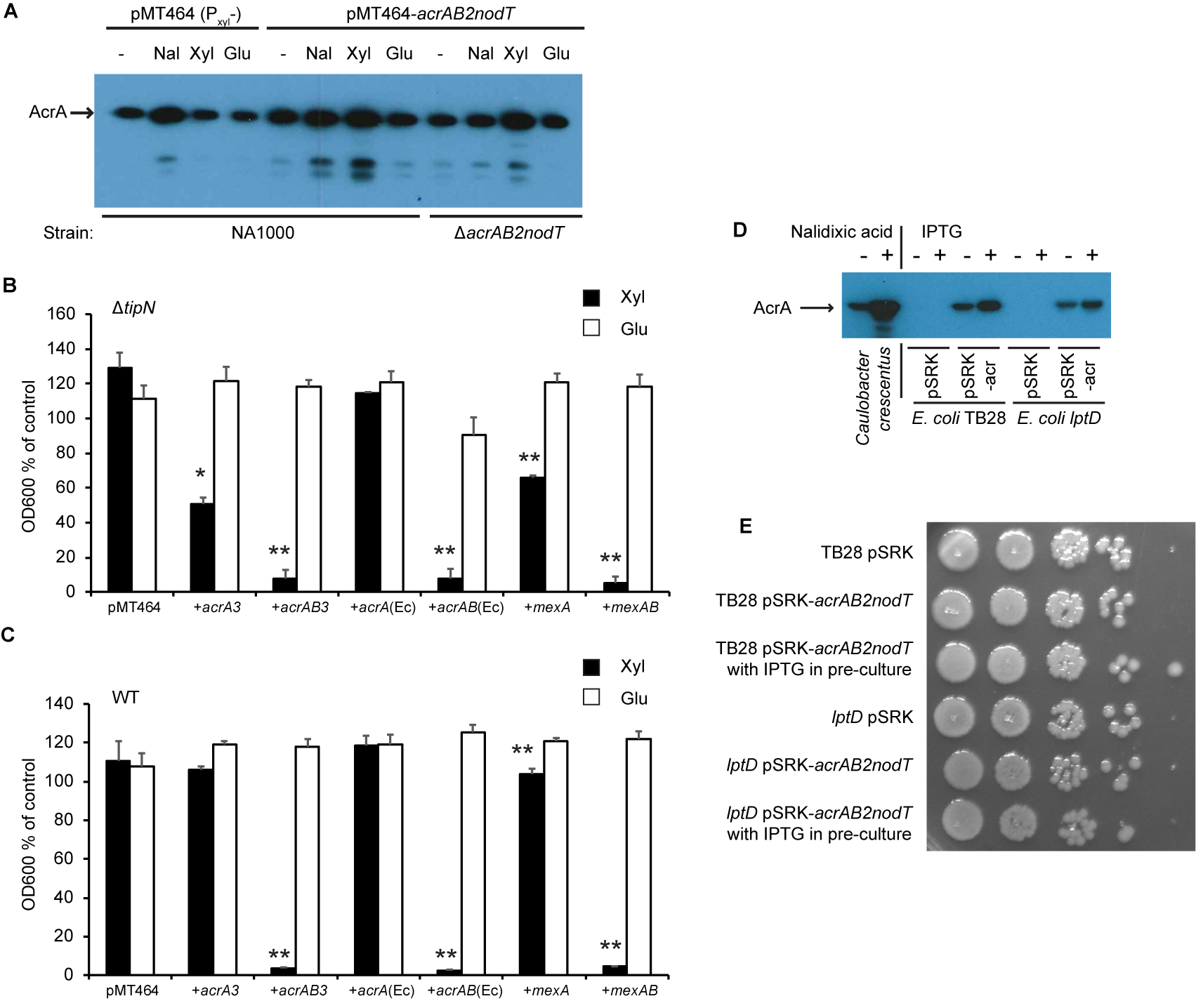

Supplement: FIG S1 [file mBio.00538-20-sf001.tif]

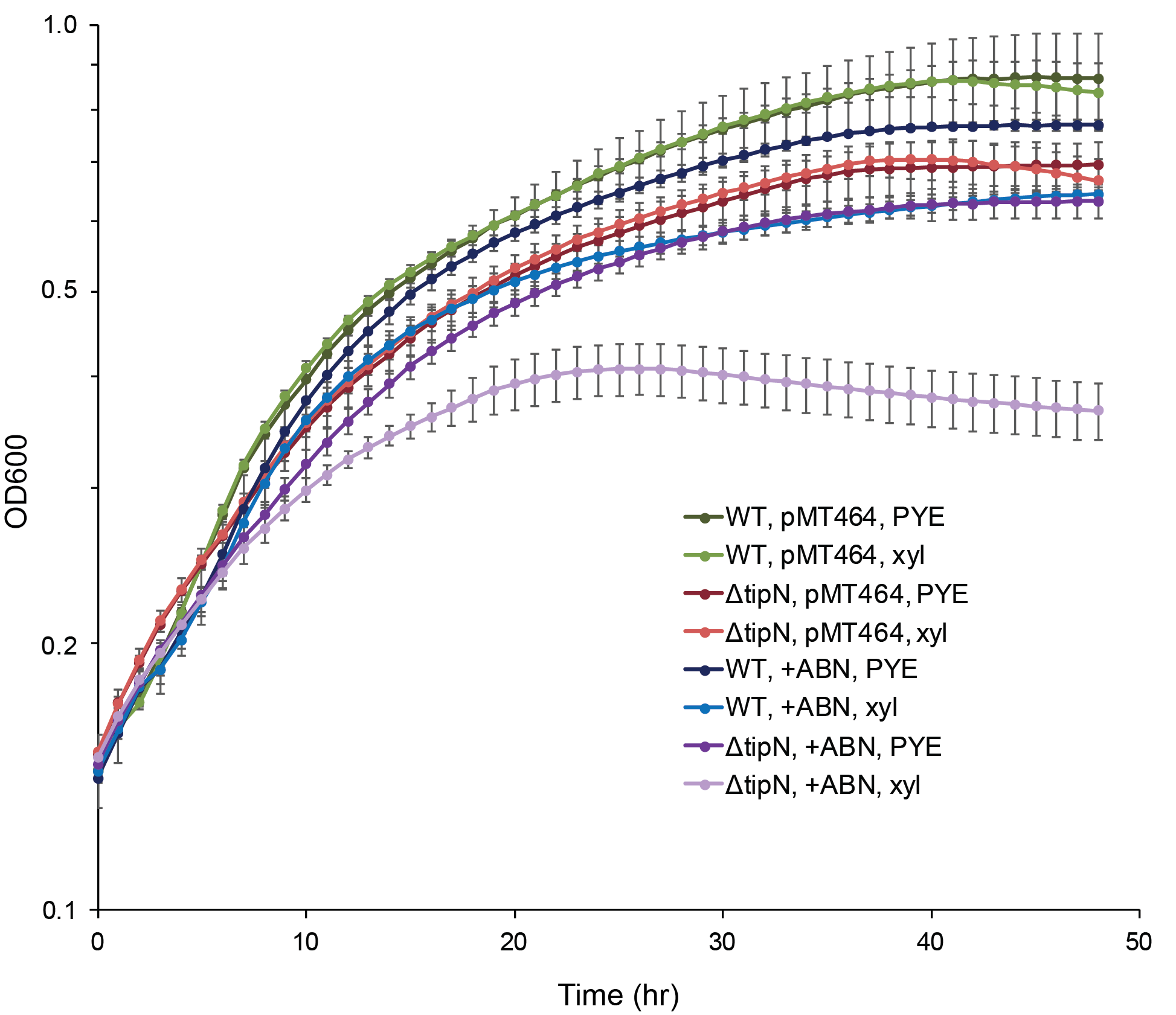

Supplement: FIG S2 [file mBio.00538-20-sf002.tif]

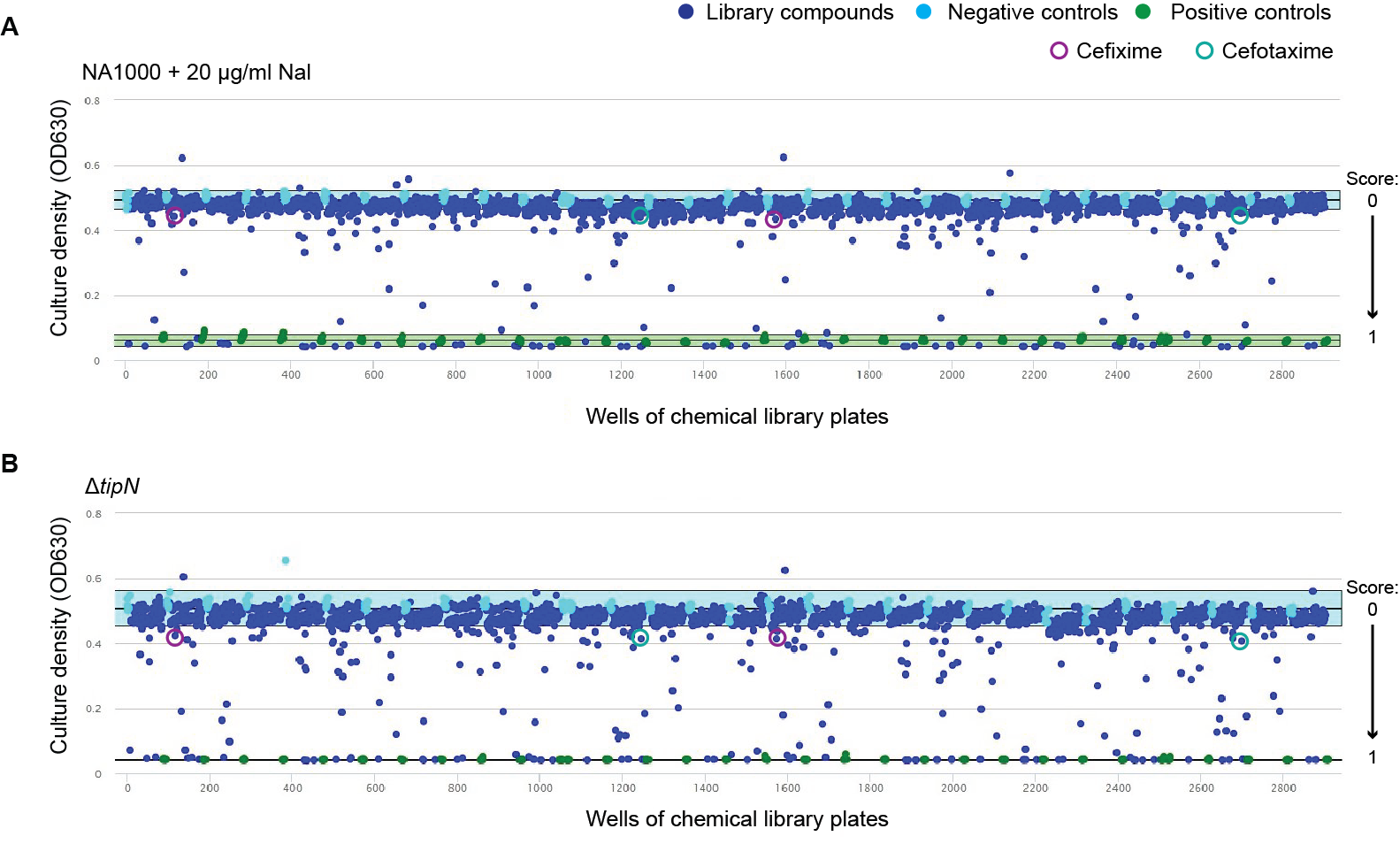

Supplement: FIG S3 [file mBio.00538-20-sf003.tif]

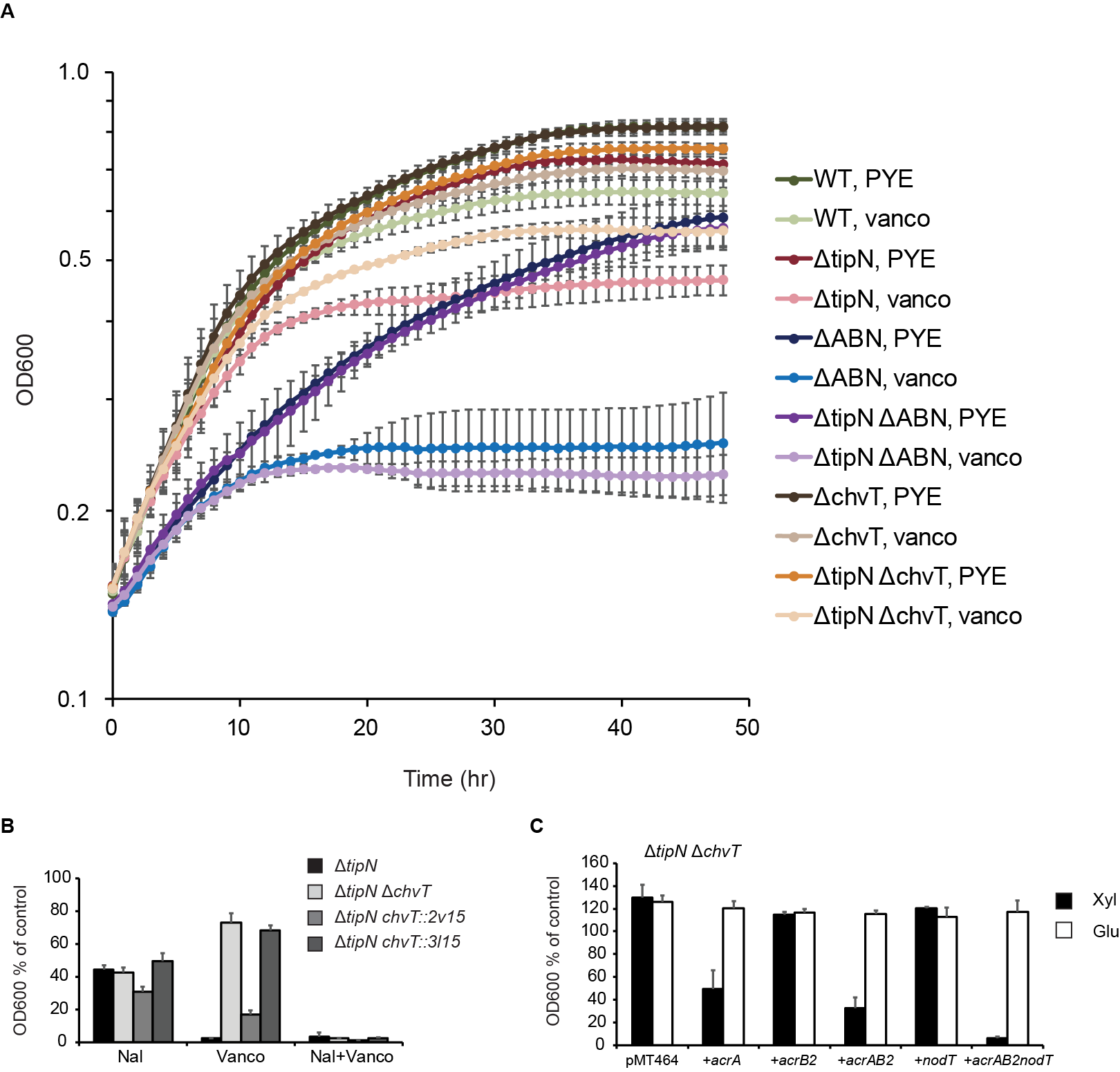

Supplement: FIG S4 [file mBio.00538-20-sf004.tif]

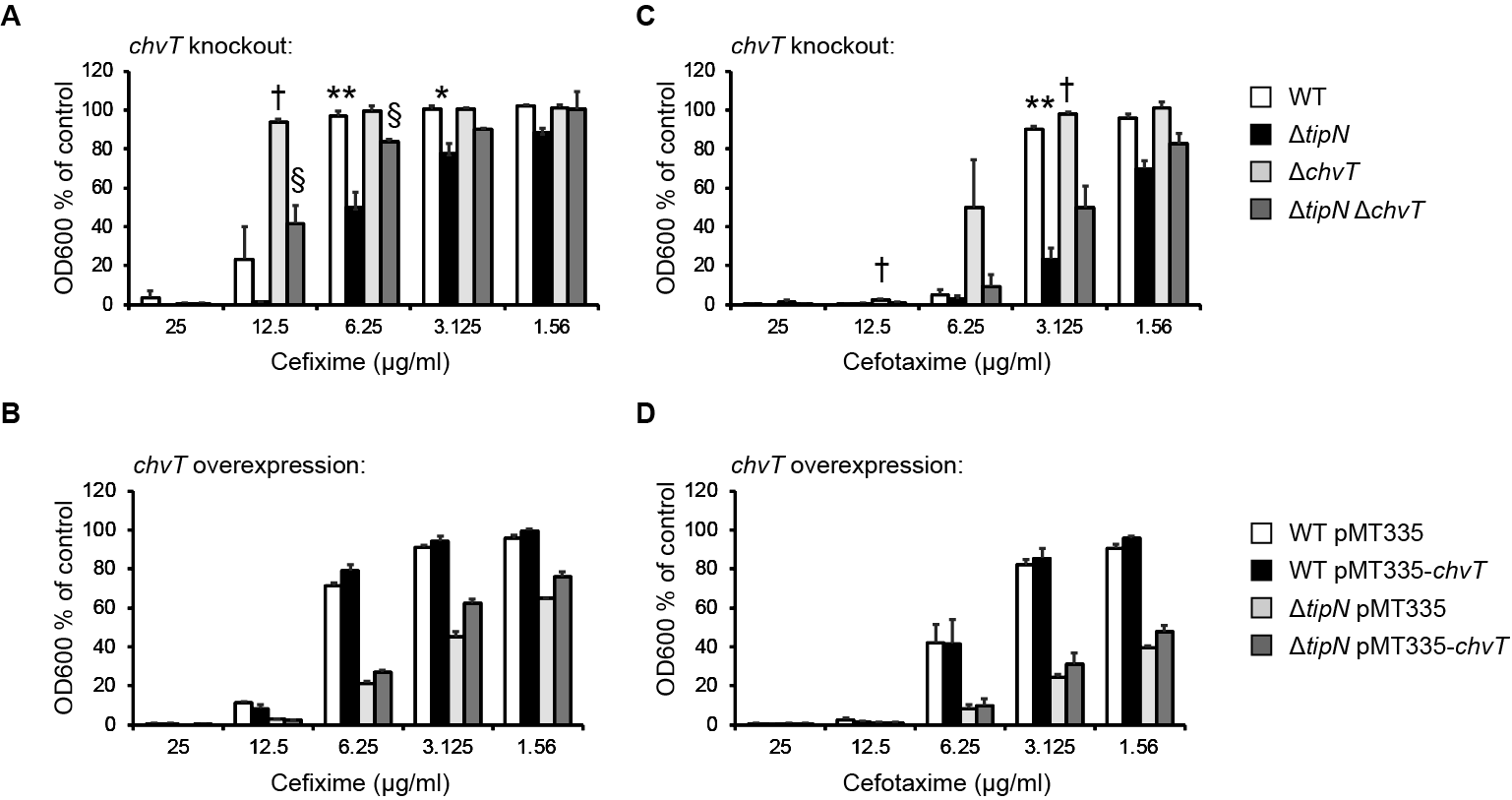

Supplement: FIG S5 [file mBio.00538-20-sf005.tif]
