# Supplementary material for: Loss of Bacterial Cell Pole Stabilization in Caulobacter crescentus Sensitizes to Outer Membrane Stress and Peptidoglycan-Directed Antibiotics
Source: mBio. 2020 May 5;11(3):e00538-20. doi: 10.1128/mBio.00538-20 (PMC7403779; doi:10.1128/mBio.00538-20)
Supplement: TABLE S1 [file mBio.00538-20-st001.docx]

**Table S1. Primers used in this study.** Restriction enzyme sites incorporated into primers for cloning purposes are underlined.

| Primer name | Sequence (5’ – 3’) |
| --- | --- |
| acrA_nosigpep_nde | AAACATATGGGCCAGAAGCCGGGCGGC |
| acrA_eco | AAAGAATTCAGCGCTGAGCTTGGGCGG |
| acrA_nde | AAACATATGCATTTCCAACGCTCGGTCGC |
| nodT_nde | AAACATATGCTGCGTAACTTCACCCTGATC |
| nodT_eco_ii | AAAGAATTCGGATGATGGTCGTCGAACTC |
| acrA3_Cc_nde | AAACATATGATTCGTAGGCACTTCTTCCTC |
| acrA3_Cc_sac | AAAGAGCTCAGCCGGCCTTGCGGTCAGC |
| acrB3_Cc_sac | AAAGAGCTCATGCGGGTGAGGTTTGCCCCTC |
| acrA_Ec_nde | AAACATATGAACAAAAACAGAGGGTTTACG |
| acrA_Ec_sac | AAAGAGCTCAAGACTTGGACTGTTCAGGC |
| acrB_Ec_sac | AAAGAGCTCAATGATGATCGACAGTATG |
| mexA_Pa_nde | AAACATATGCAACGAACGCCAGCCATG |
| mexA_Pa_sac | AAAGAGCTCAGCCCTTGCTGTCGGTTTTG |
| mexB_Pa_sac | AAAGAGCTCATTGCCCCTTTTCGACGGAC |
| 3013_nde | AAACATATGGCGTTGAAAACCAAGGTTCTC |
| 3013_nhe | AAAGCTAGCTTACATCTTGTAGTTGAAG |
| 3013_up_bam | AAAGGATCCCTTGGTTTTCAACGCCATGT |
| 3013_up_hind | AAAAAGCTTGGTCTGACCGAGGATCACAG |
| 3013_down_eco | AAAGAATTCTTTGACGCGATAACGAGGCT |
| 3013_down_bam | AAAGGATCCGGCTTCAACTACAAGATGTAAGC |
| P-3013-eco | AAAAGAATTCGTCATCGGGTGTCGAAGAGG |
| P-3013-spe | AAAAACTAGTGGTGACCACGACTTCCTCAA |
| pchvR_eco_f | AAAGAATTCTGAAATCTCGAAGAACAGCGGA |
| pchvR_xba_r | AAATCTAGACCGGAGACTTGCGCGACATG |
| pMar2xT7_Arb1_A | TACAGTTTACGAACCGAACAGGC |
| pMar2xT7_Arb1_B | GGCCAGGCCTGCAGATGATGNNNNNNNNNNGTAT |
| pMar2xT7_Arb2_A | TGTCAACTGGGTTCGTGCCTTCATCCG |
| pMar2xT7_Arb2_B | GGCCAGGCCTGCAGATGATG |
| pMar2xT7_Arb3_A | GACCGAGATAGGGTTGAGTG |
| chvI_nde_fwd | AAACATATGGCCGCGATCACGCTCATTGA |
| chvI_xba_rev | AAATCTAGAAGCGCCGCTTGGGCTCG |
