# Supplementary material for: Loss of Bacterial Cell Pole Stabilization in Caulobacter crescentus Sensitizes to Outer Membrane Stress and Peptidoglycan-Directed Antibiotics
Source: mBio. 2020 May 5;11(3):e00538-20. doi: 10.1128/mBio.00538-20 (PMC7403779; doi:10.1128/mBio.00538-20)
Supplement: TABLE S2 [file mBio.00538-20-st002.docx]

**Table S2. Plasmids used in this study.**

| Plasmid name | Description |
| --- | --- |
| pET28a | pBR322 and f1 ori, KanR, commercial vector for N-terminal His-tagged protein overexpression (Novagen) |
| pET28-*acrA* | pET28a derivative containing a truncated *acrA* allele replacing the putative signal sequence for periplasmic export with N-terminal His-tag |
| pMT335 | pBBR1 ori, rep, mob, GentR, high copy plasmid for vanillate-inducible gene expression (1) |
| pMT335-*acrA* | pMT335 derivative, P_van_-*acrA*  (2) |
| pMT335-*acrB2* | pMT335 derivative, P_van_-*acrB2* (2) |
| pMT335-*acrAB2* | pMT335 derivative, P_van_-*acrAB2*  (2) |
| pMT335-*acrAB2nodT* | pMT335 derivative, P_van_-*acrAB2nodT* |
| pMT464 | pBBR1 ori, rep, mob, KanR, high copy plasmid for xylose-inducible gene expression (1) |
| pMT464-*acrA* | pMT464 derivative, P_xyl_-*acrA* |
| pMT464-*acrB2* | pMT464 derivative, P_xyl_-*acrB2* |
| pMT464-*acrAB2* | pMT464 derivative, P_xyl_-*acrAB2* |
| pMT464-*nodT* | pMT464 derivative, P_xyl_-*nodT* |
| pMT464-*acrAB2nodT* | pMT464 derivative, P_xyl_-*acrAB2nodT* |
| pMT464-*acrA3* | pMT464 derivative, P_xyl_-*acrA3* |
| pMT464-*acrAB3* | pMT464 derivative, P_xyl_-*acrAB3* |
| pMT464-*acrA(Ec)* | pMT464 derivative, P_xyl_-*acrA* (from *E. coli* MG1655) |
| pMT464-*acrAB(Ec)* | pMT464 derivative, P_xyl_-*acrAB* (from *E. coli* MG1655) |
| pMT464-*mexA* | pMT464 derivative, P_xyl_-*mexA* (from *Pseudomonas aeruginosa* PAO1) |
| pMT464-*mexAB* | pMT464 derivative, P_xyl_-*mexAB* (from *Pseudomonas aeruginosa* PAO1) |
| pSRK-Km | pBBR1 ori, rep, mob, KanR, high copy plasmid for IPTG-inducible gene expression (3) |
| pSRK-*acrAB2nodT* | pSRK-Km derivative, P_lac_-*acrAB2nodT* |
| pMR2xT7 | λ*pir*-dependent ori, tra, AmpR, GentR, *himar1* transposon delivery vector (4) |
| pMT335-*chvT* | pMT335 derivative, P_van_-*chvT* |
| plac290 | oriV, TetR, *lacZ* transcriptional fusion vector (low copy) |
| pP*_chvT_*-lac290 | plac290 derivative with promoter of *chvT* inserted upstream of *lacZ* |
| pP*_chvT_*::1h15-lac290 | plac290 derivative with *himar1*-disrupted promoter of *chvT* inserted upstream of *lacZ* |
| pNPTS138 | colEI ori, M13 ori, oriT, KanR, *sacB*, suicide vector for in-frame deletions |
| pNPTSΔ*chvT* | pNPTS138 derivative to introduce Δ*chvT* allele |
| pP*_chvR_*-lac290 | plac290 derivative with promoter of *chvR* inserted upstream of *lacZ* |
| pMT464-*chvI* | pMT464 derivative, P_xyl_-*chvI* |
| pBVMCS-6 | pBBR1 ori, rep, mob, ChlorR, high copy plasmid for vanillate-inducible gene expression (1) |
| pKF382 | pBVMCS-6 derivative, P_van_-*chvR* (5) |

**Table S2 references**

1. Thanbichler M, Iniesta AA, Shapiro L. 2007. A comprehensive set of plasmids for vanillate- and xylose-inducible gene expression in Caulobacter crescentus. Nucleic Acids Res 35:e137.

2. Kirkpatrick CL, Viollier PH. 2014. Synthetic Interaction between the TipN Polarity Factor and an AcrAB-Family Efflux Pump Implicates Cell Polarity in Bacterial Drug Resistance. Chem Biol 21:657-65.

3. Khan SR, Gaines J, Roop RM, 2nd, Farrand SK. 2008. Broad-host-range expression vectors with tightly regulated promoters and their use to examine the influence of TraR and TraM expression on Ti plasmid quorum sensing. Appl Environ Microbiol 74:5053-62.

4. Liberati NT, Urbach JM, Miyata S, Lee DG, Drenkard E, Wu G, Villanueva J, Wei T, Ausubel FM. 2006. An ordered, nonredundant library of Pseudomonas aeruginosa strain PA14 transposon insertion mutants. Proc Natl Acad Sci U S A 103:2833-8.

5. Fröhlich KS, Förstner KU, Gitai Z. 2018. Post-transcriptional gene regulation by an Hfq-independent small RNA in Caulobacter crescentus. Nucleic Acids Research 46:10969-10982.
