# Supplementary material for: Loss of Bacterial Cell Pole Stabilization in Caulobacter crescentus Sensitizes to Outer Membrane Stress and Peptidoglycan-Directed Antibiotics
Source: mBio. 2020 May 5;11(3):e00538-20. doi: 10.1128/mBio.00538-20 (PMC7403779; doi:10.1128/mBio.00538-20)
Supplement: TABLE S3 [file mBio.00538-20-st003.docx]

**Table S3. Bacterial strains used in this study**

| *Caulobacter crescentus* strain | Description/genotype |
| --- | --- |
| NA1000 | Wild-type *Caulobacter crescentus* |
| NR1751 | Δ*tipN* |
| CLK127 | Δ*tipN* pMT335 |
| CLK885 | Δ*tipN* pMT335-*acrA* |
| CLK889 | Δ*tipN* pMT335*-acrAB2* |
| CLK997 | Δ*tipN* pMT335-*acrAB2nodT* |
| CLK716 | Δ*tipN* Δ*acrAB2nodT* |
| CLK913 | Δ*tipN* Δ*acrAB2nodT* pMT335 |
| CLK915 | Δ*tipN* Δ*acrAB2nodT* pMT335-*acrA* |
| CLK919 | Δ*tipN* Δ*acrAB2nodT* pMT335-*acrAB2* |
| CLK1001 | Δ*tipN* Δ*acrAB2nodT* pMT335-*acrAB2nodT* |
| CLK1125 | NA1000 pMT464 |
| CLK1155 | NA1000 pMT464-*acrA* |
| CLK1159 | NA1000 pMT464-*acrAB2* |
| CLK1157 | NA1000 pMT464-*acrB2* |
| CLK1161 | NA1000 pMT464-*nodT* |
| CLK1115 | NA1000 pMT464-*acrAB2nodT* |
| CLK719 | Δ*acrAB2nodT* |
| CLK1133 | Δ*acrAB2nodT* pMT464-*acrAB2nodT* |
| CLK1127 | Δ*tipN* pMT464 |
| CLK1163 | Δ*tipN* pMT464-*acrA* |
| CLK1167 | Δ*tipN* pMT464*-acrAB2* |
| CLK1165 | Δ*tipN* pMT464-*acrB2* |
| CLK1169 | Δ*tipN* pMT464-*nodT* |
| CLK1117 | Δ*tipN* pMT464-*acrAB2nodT* |
| CLK1280 | Δ*tipN* pMT464-*acrA3* |
| CLK1294 | Δ*tipN* pMT464-*acrAB3* |
| CLK1278 | Δ*tipN* pMT464-*acrA(Ec)* |
| CLK1296 | Δ*tipN* pMT464-*acrAB(Ec)* |
| CLK1282 | Δ*tipN* pMT464-*mexA* |
| CLK1284 | Δ*tipN* pMT464-*mexAB* |
| CLK1316 | NA1000 pMT464-*acrA3* |
| CLK1298 | NA1000 pMT464-*acrAB3* |
| CLk1318 | NA1000 pMT464-*acrA(Ec)* |
| CLK1300 | NA1000 pMT464-*acrAB(Ec)* |
| CLK1320 | NA1000 pMT464-*mexA* |
| CLK1302 | NA1000 pMT464-*mexAB* |
| LT1856 | NA1000 pP*_chvT_*-lac290 |
| LT1857 | NA1000 pP*_chvT_*::1h15-lac290 |
| LT1858 | Δ*tipN* pP*_chvT_*-lac290 |
| LT1859 | Δ*tipN* pP*_chvT_*::1h15-lac290 |
| CLK1535 | Δ*chvT* |
| CLK1539 | Δ*tipN* Δ*chvT* |
| CLK1557 | Δ*tipN P_chvT_::1h15* |
| CLK1559 | Δ*tipN chvT::2v15* |
| CLK1561 | Δ*tipN chvT::3l15* |
| CLK131 | NA1000 pMT335 |
| CLK1586 | NA1000 pMT335-*chvT* |
| CLK1588 | Δ*tipN* pMT335-*chvT* |
| CLK1590 | Δ*tipN* Δ*chvT* pMT464 |
| CLK1592 | Δ*tipN* Δ*chvT* pMT464-*acrA* |
| CLK1594 | Δ*tipN* Δ*chvT* pMT464-*acrAB2* |
| CLK1596 | Δ*tipN* Δ*chvT* pMT464-*acrB2* |
| CLK1598 | Δ*tipN* Δ*chvT* pMT464-*nodT* |
| CLK1600 | Δ*tipN* Δ*chvT* pMT464-*acrAB2nodT* |
| KFS-0185 | Δ*chvIG-hprK* (1) |
| CLK1581 | NA1000 pP*_chvR_*-lac290 |
| CLK1582 | Δ*tipN* pP*_chvR_*-lac290 |
| CLK1583 | Δ*chvT* pP*_chvR_*-lac290 |
| CLK1584 | Δ*tipN* Δ*chvT* pP*_chvR_*-lac290 |
| CLK1585 | Δ*chvIG-hprK* pP*_chvR_*-lac290 |
| CLK1620 | NA1000 pMT464 pP*_chvR_*-lac290 |
| CLK1621 | NA1000 pMT464-*acrAB2nodT* pP*_chvR_*-lac290 |
| CLK1622 | Δ*tipN* pMT464 pP*_chvR_*-lac290 |
| CLK1623 | Δ*tipN* pMT464-*acrAB2nodT* pP*_chvR_*-lac290 |
| CLK1648 | NA1000 pMT464-*chvI* |
| CLK1646 | NA1000 pBVMCS-6 |
| CLK1644 | NA1000 pKF382 (P_van_-*chvR*) |
| KFS-0391 | *chvT::3xFLAG* (1) |
| CLK1656 | *chvT::3xFLAG* pMT464 |
| CLK1654 | *chvT::3xFLAG* pMT464-*chvI* |
| CLK1652 | *chvT::3xFLAG* pBVMCS-6 |
| CLK1650 | *chvT::3xFLAG* pKF382 (P_van_-*chvR*) |
| *E. coli* strain | Description/genotype (source) |
| TB28 | *E. coli* MG1655 Δ[lac operon] |
| TB28 derivative | TB28 pSRK-Km |
| TB28 derivative | TB28 pSRK-*acrAB2nodT* |
| NR698 | *E. coli* MC4100 *lptD* (imp-4213 allele) (2) |
| NR698 derivative | NR698 pSRK-Km |
| NR698 derivative | NR698 pSRK-*acrAB2nodT* |

**Table S3 references**

1. Fröhlich KS, Förstner KU, Gitai Z. 2018. Post-transcriptional gene regulation by an Hfq-independent small RNA in Caulobacter crescentus. Nucleic Acids Research 46:10969-10982.

2. Ruiz N, Falcone B, Kahne D, Silhavy TJ. 2005. Chemical Conditionality: A Genetic Strategy to Probe Organelle Assembly. Cell 121:307-317.
