# Supplementary material for: Loss of Bacterial Cell Pole Stabilization in Caulobacter crescentus Sensitizes to Outer Membrane Stress and Peptidoglycan-Directed Antibiotics
Source: mBio. 2020 May 5;11(3):e00538-20. doi: 10.1128/mBio.00538-20 (PMC7403779; doi:10.1128/mBio.00538-20)
Supplement: TEXT S1 [file mBio.00538-20-s0001.docx]

**Supplemental Methods**

***Details of plasmid construction***

DNA fragments for cloning were amplified by PCR from *Caulobacter crescentus* NA1000 unless otherwise stated, using stationary phase cultures as template. PCR was performed with KOD polymerase (Novagen) or Phusion polymerase (NEB) according to the manufacturer’s instructions, using specific primers listed in Table S1, and products were purified by agarose gel electrophoresis. Cloning of the correct region was confirmed by DNA sequencing, and plasmid stocks were maintained in *E. coli* EC100 (Epicentre) or TOP10 (Invitrogen). Plasmid characteristics are listed in Table S2.

For overexpression and purification of soluble AcrA protein, part of the *acrA* gene was amplified with primers acrA_nosigpep_nde and acrA_eco to generate an N-terminal truncated gene lacking the first 23 codons, which contain a signal sequence for export to the periplasm and convert the Cys24 codon to Met as part of the *Nde*I restriction site. This fragment was digested with *Nde*I and *EcoR*I and ligated into correspondingly digested pET28a, fusing the truncated *acrA* gene to a N-terminal His-tag.

For overexpression of *acrA*, *acrB2* and *acrAB2* from the xylose-inducible promoter of pMT464, fragments corresponding to these coding regions were excised from pMT335-*acrA*, pMT335-*acrB2* and pMT335-*acrAB2* by *Nde*I/*Eco*RI digestion and separated from the pMT335 vector by gel electrophoresis. They were then cloned as *Nde*I/*Eco*RI fragments into correspondingly digested pMT464 to form pMT464-*acrA*, pMT464-*acrB2* and pMT464-*acrAB2*, respectively. For overexpression of the *nodT* gene and the *acrAB2nodT* operon, fragments were amplified by PCR using primers nodT_nde and nodT_eco_ii (for *nodT* alone) and acrA_nde and nodT_eco_ii (for the operon), digested with *Nde*I and *Eco*RI and ligated into correspondingly digested pMT464 to give pMT464-*nodT* and pMT464-*acrAB2nodT*. In parallel, the *acrAB2nodT* operon fragment was also ligated into *Nde*I/*Eco*RI-digested pMT335 to give pMT335-*acrAB2nodT*. For overexpression of the alternative *Caulobacter crescentus* efflux pump components *acrA3* (CCNA_ 02161) and *acrAB3* (CCNA_02161 and 02162), fragments corresponding to these coding regions were amplified using primers acrA3_Cc_nde and acrA3_Cc_sac (for *acrA3*) and acrA3_Cc_nde and acrB3_Cc_sac (for *acrAB3*), digested with *Nde*I and *Sac*I and ligated into correspondingly digested pMT464 to give pMT464-*acrA3* and pMT464-*acrAB3*. The pMT464-*chvI* overexpression plasmid was constructed by amplification of *chvI* with primers chvI_nde_fwd and chvI_xba_rev, digestion with *Nde*I and *Xba*I and ligation into correspondingly digested pMT464.

To construct the heterologous overexpression plasmids pMT464-*acrA*(Ec) and pMT464-*acrAB*(Ec), primers acrA_Ec_nde and acrA_Ec_sac (for *acrA*) and acrA_Ec_nde and acrB_Ec_sac (for *acrAB*) were used to amplify these genes using *E. coli* MG1655 genomic DNA as PCR template. Fragments were digested with *Nde*I and *Sac*I and ligated into correspondingly digested pMT464 to give pMT464-*acrA(Ec)* and pMT464-*acrAB(Ec)*. The same procedure was used to construct the heterologous overexpression plasmids pMT464-*mexA* and pMT464-*mexAB*, using *Pseudomonas aeruginosa* PAO1 genomic DNA as PCR template, primers mexA_Pa_nde and mexA_Pa_sac to amplify the *mexA* gene and primers mexA_Pa_nde and mexB_Pa_sac to amplify the *mexAB* genes. To transfer the *Caulobacter crescentus* *acrAB2nodT* operon into the vector pSRK-Km for heterologous overexpression in *E. coli*, pMT335-*acrAB2nodT* was digested with *Nde*I and *Xba*I and the *acrAB2nodT* fragment separated from the pMT335 vector backbone by gel electrophoresis. The fragment was ligated into *Nde*I/*Xba*I-digested pSRK-Km to give pSRK-*acrAB2nodT*.

For overexpression of *chvT* from the vanillate-inducible promoter, the coding region of *chvT* was amplified with primers 3013_nde and 3013_nhe, digested with *Nde*I and *Nhe*I and ligated into correspondingly digested pMT335 to give pMT335-*chvT*. To construct the *chvT* knockout plasmid pNPTSΔ*chvT*, two homology drivers were amplified from the regions upstream and downstream of the *chvT* gene. The 5’ homology driver consisted of 623 bp containing the first 6 codons of the *chvT* coding sequence, amplified with 3013_up_bam and 3013_up_hind, while the 3’ homology driver consisted of 689 bp containing the last 6 codons of the *chvT* coding region, amplified with 3013_down_eco and 3013_down_bam. These were digested with *Hin*dIII/*Bam*HI and *Bam*HI/*Eco*RI respectively, and ligated simultaneously into *Eco*RI/*Hin*dIII digested pNPTS138 to create pNPTSΔ*chvT*.

Transcriptional fusions of the intact or *himar1-*disrupted *chvT* promoter to *lacZ* were made by amplifying the *chvT* promoter region with primers P-3013-eco and P-3013-spe using either WT *Caulobacter* or the Δ*tipN P_chvT_::1h15* transposon mutant strain as template, digesting the resulting products with *Eco*RI/*Spe*I and ligating them into *Eco*RI/*Xba*I-digested plac290 to give pP*_chvT_*-lac290 and pP*_chvT_*::1h15-lac290, respectively. Transcriptional fusion of the *chvR* promoter to *lacZ* was made by amplifying the *chvR* promoter region with pchvR_eco_f and pchvR_xba_r, digesting with *Eco*RI and *Xba*I and ligating into correspondingly digested plac290 to give pP*_chvR_*-lac290.

***AcrA protein purification and western blotting***

His6-AcrA was overexpressed by addition of 1 mM IPTG from pET28-*acrA* in *E. coli* Rosetta and purified under native conditions by Ni-NTA chelate chromatography. Purified His6-AcrA protein was used to immunize rabbits (Josman LLC, USA) for antiserum production. Cell extracts were prepared from mid-exponential phase cultures normalized to culture density, electrophoresed in 15% SDS-polyacrylamide gels and the proteins transferred to PVDF membranes. The anti-AcrA antiserum was diluted to 1/50’000 and detected by a horseradish peroxidase-coupled secondary antibody with chemiluminescent substrate. Anti-FLAG primary antibody (mouse monoclonal, Merck) was used at a 1/500’000 dilution for detection of ChvT::3xFLAG. Anti-MucR antiserum (1) was used as a loading control at 1/10’000 dilution.

***End-point growth assays***

To measure relative growth in liquid culture with antibiotics or during efflux pump gene overexpression, *Caulobacter* strains were grown to stationary phase, culture density (OD600) was measured and the starter cultures were diluted to a final density of OD600 = 0.001 (equivalent to 10^6^ cells/ml) into 5 ml PYE containing the antibiotic to be tested along with other antibiotic selection markers for plasmids. Where appropriate, 50 μM vanillate to induce gene expression from pMT335 derivatives, and 0.3% xylose to induce or 0.2% glucose to repress gene expression from pMT464 derivatives, was added. Cultures were grown for 20hr at 30°C (to early stationary phase) and the OD600 measured. Data are expressed throughout as a percentage of the OD600 of a control culture of each strain, including plasmid selection markers where necessary but without any other supplement. All graphs show mean ± SEM from three independent experiments.

***Cfu/ml to OD600 calibration***

Stationary phase cultures were diluted 1/1000 into PYE containing no drug, 20 µg/ml Nal or 15 µg/ml Vanco and incubated at 30°C for 24hr. The OD600 of each culture was measured, then the culture was serially (tenfold) diluted in PYE and samples from the 10^-5^ and 10^-6^ dilutions of control or Nal cultures, or 10^-4^ and 10^-5^ dilutions of Vanco cultures, plated out on PYE agar without antibiotic and incubated at 30°C for 3 days. Colonies were counted and the resulting values of cfu/ml in the cultures normalised by the culture OD600, to give cfu/ml values equivalent to those found in a culture with an OD600 value of 1. The graph shows mean ± SD from three independent experiments.

***Kinetic growth assays***

Growth kinetics were measured in a temperature-controlled Synergy H1 multimode plate reader (Biotek) at OD600 during 48hr incubation at 30°C. Starter cultures of *Caulobacter* strains were grown to stationary phase in PYE containing appropriate antibiotics for plasmid selection if necessary, then diluted and normalised to OD600 = 0.1. Vancomycin, xylose or glucose was supplemented as specified in the Supplemental Figure legends and 200 μl of the diluted cultures were transferred into duplicate wells of a clear flat-bottomed 96 well plate. OD600 was read once per hour with plate agitation for 15 seconds before measurement. All graphs show mean ± SD for all data points of three independent experiments.

***Efficiency of plating assays***

Resistance to vancomycin, cefixime or cefotaxime was assayed by dilution spot plating. Cultures of strains to be tested were grown overnight to stationary phase, then inoculated into new medium to grow to mid-exponential phase. Culture density was measured, normalised to the OD600 of the least dense culture (OD600=0.5 or less), serially (tenfold) diluted in PYE to 10^-6^ and 5 µl spotted onto plates containing PYE medium with no drug, 15 µg/ml vancomycin, 5 µg/ml cefixime or 5 µg/ml cefotaxime. Plates were imaged after 3 days growth at 30°C. Images are representative of three independent biological replicates. For the assays including the pMT464-*chvI* or the pKF382 (P_van_-*chvR*) overexpression constructs, 0.3% xylose (for pMT464-*chvI* and corresponding empty vector control) or 50 µM vanillate (for pKF382 and corresponding empty vector control) were added to the plates in addition to the antibiotics.

***Transposon mutant library generation, selection of vancomycin resistant mutants and mapping of transposon insertions***

The transposon mutant pooled library was created by electroporation of the transposon-bearing plasmid pMR2xT7 into *Caulobacter crescentus* Δ*tipN* followed by selection on gentamicin. After incubation at 30°C for 4 days, approximately 20’000 individual colonies were collected into a final volume of 13 ml PYE and stored at -80°C in 1 ml aliquots containing 10% DMSO. For selection of Vanco resistant clones by enrichment culture, three overnight cultures were set up from separate frozen aliquots. Upon reaching stationary phase they were diluted 1/1000 into PYE containing 15 μg/ml Vanco and incubated at 30°C for 24 hours. 100 μl aliquots were plated on solid PYE media containing 1 μg/ml gentamicin and 15 μg/ml Vanco and incubated at 30°C for 3 days. Single colonies were picked and re-streaked twice to new PYE plates with these concentrations of gentamicin and Vanco to ensure stability of the Vanco resistance. Cultures of these re-streaked clones were used as template for transposon mapping by 2-step arbitrary PCR as in (2). Briefly, the first arbitrary PCR reaction was performed in a total volume of 25 μl containing 2 μl overnight culture of the transposon mutant strains as template, 1 ng/μl of each of primers pMar2xT7_Arb1_A and pMar2xT7_Arb1_B, 2.5 μM dNTPs, 10% DMSO and 1.25 U Taq polymerase. 5 μl of this first PCR reaction was used as template for the second arbitrary PCR reaction which contained identical dNTP, DMSO and Taq concentrations and 1 ng/μl of each of primers pMar2xT7_Arb2_A and pMar2xT7_Arb2_B, in 25 μl. PCR products from these reactions were purified by agarose gel electrophoresis and sequenced with the nested sequencing primer pMar2xT7_Arb3_A. The three isolated clones with mutations in or near *chvT* (1h15, 2v15 and 3l15) had the transposon insertions backcrossed into the Δ*tipN* strain by ΦCr30 bacteriophage transduction to confirm dependence of Vanco resistance on the transposon insertions.

**References**

1. Fumeaux C, Radhakrishnan SK, Ardissone S, Theraulaz L, Frandi A, Martins D, Nesper J, Abel S, Jenal U, Viollier PH. 2014. Cell cycle transition from S-phase to G1 in Caulobacter is mediated by ancestral virulence regulators. Nat Commun 5:4081.

2. Liberati NT, Urbach JM, Miyata S, Lee DG, Drenkard E, Wu G, Villanueva J, Wei T, Ausubel FM. 2006. An ordered, nonredundant library of Pseudomonas aeruginosa strain PA14 transposon insertion mutants. Proc Natl Acad Sci U S A 103:2833-8.
